# Molecular Structure Tailoring of Organic Spacers for High-Performance Ruddlesden–Popper Perovskite Solar Cells

**DOI:** 10.1007/s40820-024-01500-7

**Published:** 2024-10-10

**Authors:** Pengyun Liu, Xuejin Li, Tonghui Cai, Wei Xing, Naitao Yang, Hamidreza Arandiyan, Zongping Shao, Shaobin Wang, Shaomin Liu

**Affiliations:** 1 School of Materials Science and Engineering, State Key Laboratory of Heavy Oil Processing, China University of Petroleum (East China), Qingdao, 266580 People’s Republic of China; 2 School of Chemistry and Chemical Engineering, Shandong University of Technology, Zibo, 255049 People’s Republic of China; 3 Centre for Applied Materials and Industrial Chemistry (CAMIC), School of Science, RMIT University, Melbourne, Vic 3000 Australia; 4 WA School of Mines: Minerals, Energy and Chemical Engineering (WASM-MECE), Curtin University, Perth, WA 6102 Australia; 5 School of Chemical Engineering, The University of Adelaide, Adelaide, SA 5005 Australia; 6 School of Engineering, Great Bay University, Dongguan, 523000 People’s Republic of China

**Keywords:** Ruddlesden–Popper perovskites, Low-dimensional perovskite solar cells, Organic spacers, Molecular structure, Design strategies

## Abstract

Layer-structured Ruddlesden–Popper (RP) perovskites (RPPs) with decent stability have captured the imagination of the photovoltaic research community and bring hope for boosting the development of perovskite solar cell (PSC) technology. However, two-dimensional (2D) or quasi-2D RP PSCs are encountered with some challenges of the large exciton binding energy, blocked charge transport and poor film quality, which restrict their photovoltaic performance. Fortunately, these issues can be readily resolved by rationally designing spacer cations of RPPs. This review mainly focuses on how to design the molecular structures of organic spacers and aims to endow RPPs with outstanding photovoltaic applications. We firstly elucidated the important roles of organic spacers in impacting crystallization kinetics, charge transporting ability and stability of RPPs. Then we brought three aspects to attention for designing organic spacers. Finally, we presented the specific molecular structure design strategies for organic spacers of RPPs aiming to improve photovoltaic performance of RP PSCs. These proposed strategies in this review will provide new avenues to develop novel organic spacers for RPPs and advance the development of RPP photovoltaic technology for future applications.

## Highlights

- Organic spacers in Ruddlesden–Popper (RP) perovskites play a vital role in tuning crystallization, charge transport and photovoltaic performance for RP perovskite solar cells (PSCs).
- Fundamental understanding of the functions of molecular structure of organic spacers is the prerequisite to design high-performance PSCs.
- This review proposes practical design strategies in seeking RP molecular structure to maximize its photovoltaic performance for PSCs.

## Graphical abstract

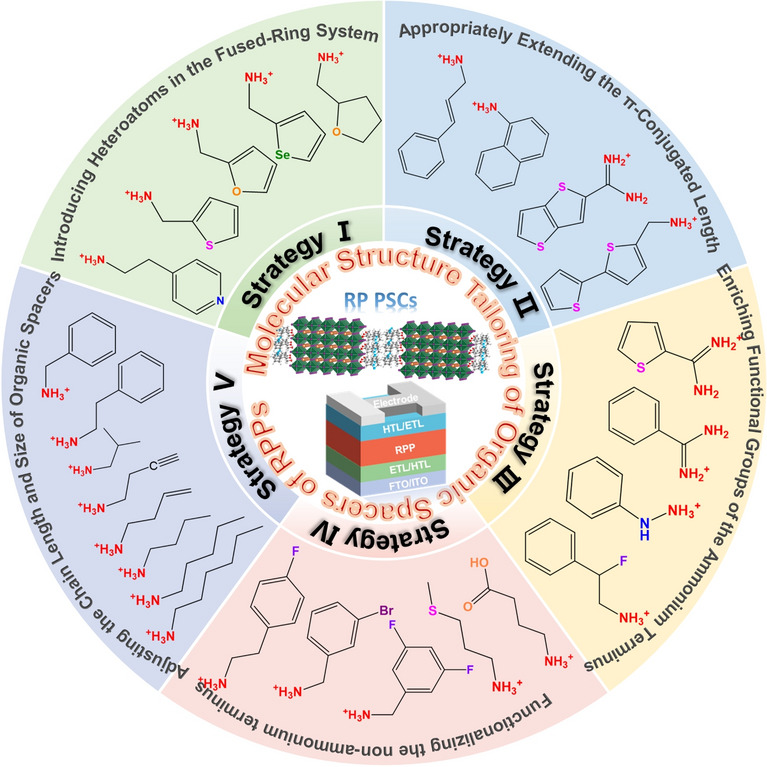

## Introduction

Two-dimensional (2D) Ruddlesden−Popper perovskites (RPPs) have emerged as promising semiconductor materials in perovskite solar cells (PSCs) due to their superior stability and tunable optoelectronic properties [1–4]. 2D RPPs are in layered crystal structure with a general A’_2_A_n-1_B_n_X_3n+1_ stoichiometry, where A’ stands for the bulky monovalent spacer cations such as n-butylammonium (n-BA), phenylmethylammonium (PMA) and phenylethylammonium (PEA), A means small monovalent organic ammonium ions like methylammonium ion (CH_3_NH_3_^+^, MA), formamidinium ion (CH(NH_2_)_2_^+^, FA) or inorganic Cs^+^, B represents lead ions (Pb^2+^) or tin ions (Sn^2+^), and X refers to halide ions including I^−^, Br^−^ and Cl^−^ [5, 6]. The A’ spacer cations separate one set of 3D perovskite layers from next and connect the inorganic [BX_6_]^4−^ octahedron framework via ionic and hydrogen bonding (HB) interactions [7, 8]. *n* denotes the number of consecutive corner-shared inorganic [BX_6_]^4−^ octahedron layers in one set of 3D perovskite layer [9–12]. There are two layers of organic spacers between adjacent two sets of inorganic layers. The non-ammonium terminus of the bilayer of organic spacers interacts each other by the weak Van der Waals (VdW) force, leading to the existence of VdW gap between them [13].

2D RPPs have been widely explored both as a protective/passivation layer for 3D PSCs and as a complementary photovoltaic technology [14–18]. The formation of 2D RPPs on the surface of 3D perovskite layers can passivate defects, protect vulnerable 3D perovskites from water invasion and enhance stability under challenging environmental conditions, enabling 3D PSCs to achieve high power conversion efficiency (PCE) over 25% and improved long-term stability [19–25]. To further address the critical issue of the instability of 3D PSCs, RPPs have been directly utilized as light-absorbing materials to develop 2D or quasi-2D RP PSCs, which exhibit prominent stability. Unluckily, RP PSCs are encountered with serious issues to realize high PCE [7, 26–28]. These challenges include three major aspects as below. (1) RPPs possess high exciton binding energy (*E*_*b*_) of several hundred electron volts, restraining the separation of excitons to free charges [29]. (2) The organic layers and inorganic layers suffer from large dielectric mismatch, which exacerbates exciton confinement and is unfavorable for charge transporting [30]. The 2D electronic structure and dielectric confinement also make RPPs featured with a large bandgap (*E*_*g*_), limiting the light absorption range [31]. (3) The solution method-prepared RPP films bear low crystallinity and various *n*-value phase compositions with irregular distribution and crystallization direction, aggravating disorder in energy states and causing severe local recombination during the charge transport [32–34].

To address these challenges and improve the PCE of RP PSCs, numerous efforts such as additive engineering [35–39], antisolvent and solvent engineering [40–42], interface engineering [43–46], composition engineering [47–49] and preparation technique regulation [50, 51] have been devoted to optimizing film quality and ameliorating charge transport properties. For example, Zhang et al. used petroleum ether (PE) as a green antisolvent to prepare high-quality BA_2_FA_3_Pb_4_I_13_ films with large grain size and reduced defects, contributing to a PCE of 17.42% [52]. In another case, zwitterionic n-tert-butyl-α-phenylnitrone (PBN) was employed to modify the surface of PEA_2_MA_4_Pb_5_I_16_ films, which could passivate defects and simultaneously optimize crystal growth orientations of PEA_2_MA_4_Pb_5_I_16_ RPPs, consequently resulting in a high PCE of 20.05% [53]. In spite of the improved PCE of RP PSCs, these strategies cannot tune the *E*_*b*_ and improve the dielectric confinement of RPPs from the source, thereby limiting the further enhancement of RP PSC performance. Given that these issues are mainly caused by the appearance of organic spacers and closely related to their molecular structures, organic spacer designing engineering represents a promising way to battle all these shortcomings and improve the efficiency of RP PSCs. In this scenario, the key lies in developing various spacer cations by effective molecular structure design strategies to flexibly tune the inherent electronic properties of RPPs and optimize the film quality [54, 55]. A variety of novel spacer cations such as 3,5-difluorobenzylamine (DF-BZA) [56], glycine ethyl ester (Gly-E) [57], 3,3-difluoroazetidine (3,3-DFAz) [58], and *β*-​fluorophenylethanamine (*β*-​FPEA) [59] have been developed for high-efficiency RP PSCs. These newly designed organic spacers can not only tune the crystallization kinetics to form high-quality RPP films but also reduce *E*_*b*_ and the dielectric mismatch between organic and inorganic layers. Very recently, Gly-E-based RP PSCs have achieved a PCE up to 21.60%, one of the highest PCEs among all reported 2D RP PSCs (n ≤ 6) [57].

Some researchers have particularly highlighted the importance of organic spacer engineering in improving the photovoltaic performance of RPPs when reviewing the recent progress in this area [60–64]. Yet, the explicit molecular structure design strategies of organic spacers for improved RP PSCs are still lacking. Instead, this review focuses on how to rationally design organic spacers to enhance the photovoltaic performance of RP PSCs. In this review, we briefly introduce several important roles of organic spacers of RPPs in impacting crystallization process, charge transport and stability of RPPs. In particular, more emphasis is placed on the clarification of how organic spacers play such roles and affect these aspects. Then, the main concern is the design of organic spacers. We firstly point out some important factors that need to be considered when designing organic spacers. Subsequently, we present specific molecular structure design strategies for organic spacers of RPPs with the main aim to enhance the RP PSC performance. Finally, future directions which may advance the development of RP PSCs are advised.

## Roles of Organic Spacers

### Manipulating Crystallization Kinetics

#### Influence of Structure Flexibility or Rigidity of Organic Spacers

It is well known that the film quality of RPP films is decisive for photovoltaic performance of RP PSCs. Increasing researches have verified that spacer cations play an imperative role in manipulating the crystallization kinetics and affecting the RPP film quality [65, 66]. Firstly, the structure flexibility/rigidity of spacer cations exerts an influence on the film formation process. Saturated long-chain alkylammonium spacer cations with superior structure flexibility can facilitate the precursor assembly and contribute to the orientated growth of perovskite crystals, while unsaturated aromatic ammonium spacer cations with rigid structure is unfavorable for the crystallization and often lead to poor crystallinity. For example, under the similar preparation conditions, the BA_2_MA_3_Pb_4_I_13_ film was observed to exhibit more obvious (111) plane-oriented crystallization than PEA_2_MA_3_Pb_4_I_13_ film. Replacing the PEA spacer with BA can induce the higher crystallization orientation of 2D perovskites, thereby improving the film quality of PEA-based RPP films [67]. As a matter of fact, the advantage of BA spacer in promoting precursor assembly enables them a good precursor colloid chemistry and film quality regulator for many binary spacer cations (such as BA-PEA [68], BA-4-phenylbutan-1-aminium (PBA) [69], and BA-2,2,2-trifluoroethylamine (F3-EA) [70]) RPPs.

#### Influence of Size and Chain Length of Organic Spacers

Apart from the influence of structure flexibility or rigidity, the size and chain length of spacer cations affect the crystallization process of RPP films. The appropriate size and chain length of organic spacers can aid to optimize RPP film crystallinity and morphology as well as to tune the crystallization and growth orientation for RPP crystals [71]. In 2017, Zhang et al. found that short branched-chain butylamine (iso-BA) cation as spacer conferred the RPP film with higher crystallization and more preferential out-of-plane crystallization orientation under room temperature processing conditions than the typical linear chain BA spacer. In addition to iso-BA, short branched-chain guanidinium (GA) also displays superiority in enhancing the film quality and inducing the out-of-plane crystallization orientation. In most instances, GA is more suitable to serve as an alternating cation in the interlayer space (ACI) of perovskites due to its smaller ionic radius rather than a unary spacer cation for RPPs [72]. Interestingly, using GA as a secondary spacer cation in RPPs has been reported to markedly improve the crystallinity and crystallization direction of aromatic ammonium-based RPPs [73–75]. For example, introducing suitable amount of GA to replace the 4-fluorophenethylammonium (4F-PEA) spacer cation induced high-quality RPP films with superior vertical alignment and large-sized crystal grains [73]. However, the specific influencing mechanism of short branched-chain spacer cations on the crystallization process has not been investigated in these studies. We conjecture that this may link with not only the size and chain length of organic spacers but also their spatial configuration which causes different molecular interactions between organic spacers.

**Fig. 1:**
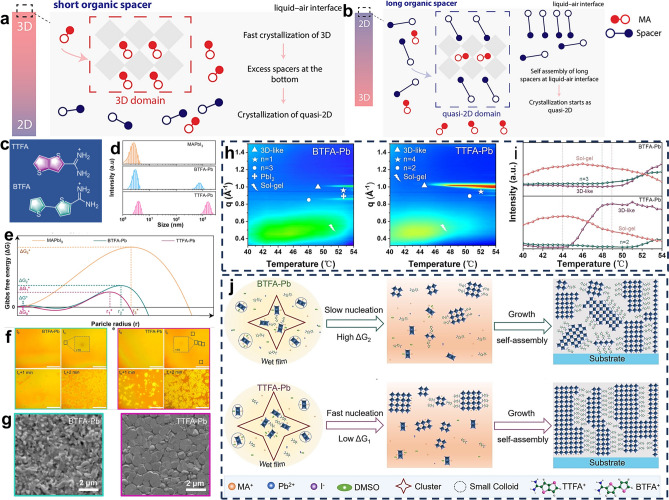
Schematic diagram illustrating the crystallization mechanism of **a** short-chain spacer and **b** long-chain spacer-based RPP films. Reproduced with permission [76]. Copyright 2024, American Chemical Society. **c** Chemical structure of TTFA and BTFA spacers. **d** DLS measurement results for of MAPbI_3_, TTFA-Pb, BTFA-Pb precursor solutions. **e** Schematic free energy profiles for TTFA-Pb, BTFA-Pb 2D RPPs. **f** *In situ* optical microscopy images showing the crystal growth process of BTFA-Pb and TTFA-Pb films at 50 °C (the scale bar is 100 nm). **g** SEM images of BTFA-Pb and TTFA-Pb films. **h** *In situ* GIWAXS results during the heating from 40 to 54 °C for BTFA-Pb and TTFA-Pb films. **i** Intensity evolution of perovskite, sol–gel and low-n-value phase extracted from *in situ *GIWAXS. **j** Schematic illustration of the crystallization process of BTFA-Pb and TTFA-Pb RP films. Reproduced with permission [83]. Copyright 2023, Wiley–VCH GmbH

The research carried out by Wu et al. may confirm our speculation [71]. They proposed a strategy via the molecular VdW interaction to regulate the crystallization process and quantum-confined behaviors by comparing the crystallization properties and film quality among different chain length alkylammonium spacers (ethylamine, EA to hexylamine, HA) for 2D (A’)_2_(MA)_3_Pb_4_I_13_ RPPs. The results demonstrate that the longer chain spacer cations with stronger VdW forces between themselves promoted aggregations in the perovskite precursor, which could modify the crystallization toward high-quality 2D RPP films with appropriate n-value phase composition [71]. In another case, the alkyl chain length of organic spacers was evidenced to influence the formation of regular- and reverse-graded quasi-2D RPP thin films [76]. The regular-graded quasi-2D RPP film is featured with 2D phases located at the bottom of the film and 3D phases at the top. *In situ* optical absorption measurements combining with photoluminescence (PL) spectroscopy illustrate that crystallization starts with the quasi-2D phases for long-chain spacers and the 3D phase for short-chain spacers. For both of them, crystallization begins at the liquid-N_2_ interfaces where the solid concentration increases with the solvent evaporating. Due to the tendency of long polar alkyl chains to accumulate at the liquid − N_2_ interface and the increasing VdW interactions between long alkyl chain, the spacers with long alkyl chains favored reverse-graded films, while the spacers with short alkyl chains resulted in regular-graded films. The proposed crystallization mechanism is given in Fig. 1a, b [76].

#### Influence of Functional Groups Appearing in Organic Spacers

Moreover, functional groups appearing in organic spacers are closely related to the nucleation and crystallization process of RPP films. Numerous newly-developed organic spacers such as DF-BZA 56, 3,3-DFAz [58], 3-guanidinopropanoic acid (GPA) [77], γ-aminobutyric acid (GABA) [78], glycine (Gly) [79], 2-(methylthio)ethylamine (MTEA) [80], and 4-(aminoethyl)pyridine (4-AEP) [81] with special functional groups such as -F, -COOH, -S-, and pyridine groups have been reported to create high-quality RPP films with high crystallinity, good crystallization orientation and decent phase composition. Noteworthy that these special functional groups of spacer cations may engage in managing the crystallization behavior of RPPs in different manners.

On the one hand, these functional groups can modulate the crystallization kinetics via strengthening intermolecular interactions between spacer cations, which affect the colloid chemistry of precursor solutions and the crystal growth process [82]. The enhanced intermolecular interactions between spacer cations can drive the formation of larger clusters serving as nucleation sites to form the perovskite crystal nucleus. Larger clusters tend to expedite nucleation process, thereby giving rise to high-quality crystals with large size and high crystallinity [78, 80, 83]. Additionally, the larger particle/cluster size in the precursor solutions will induce the particularity of phase distribution. Specifically, the strong inter-spacer interactions will restrict the coordination ability between organic spacer and [PbX_6_]^4−^ octahedra, which favors the formation of large-n phases. For example, the strong HB interactions between GABA spacers gave rise to narrower phase composition with more favorable large-n phases and even 3D perovskites instead of small-n phases [78]. Moreover, organic spacers with larger intermolecular interactions in between themselves are more likely to self-assembly into regular vertically-oriented crystals, which is favorable for charge transportation [80, 83]. For instance, both the HB interactions between GABA and GPA spacers and S…S interactions between MTEA spacers afford 2D RPPs with vertically orientated growth to the substrate [77, 78, 80].

On the other hand, some functional groups such as -C = O and pyridine groups with lone-pair electrons can regulate the crystallization process via strong interactions with Pb^2+^ in the precursor solution, which can result in the formation of intermediates during the film formation process. As for 4-AEP spacers, the N atoms on the pyridine ring could coordinate with Pb^2+^ to produce intermediates, which retards the crystallization rate of 2D perovskites and further control the growth process of RPP crystals [81]. In the case of Gly spacers, the strong coordination interaction between -C = O and Pb^2+^ is regarded to form the nucleation center around spacer cations and foster the faster and better perovskite layer growth [79].

#### Characterization of Crystallization Process of RPP Films

To probe the nucleation and crystallization process of RPP films and identify the influence of organic spacers on the film formation process, a series of characterization methods have been jointly utilized. Dynamic light scattering (DLS) measurements and *in situ* optical microscopy images are powerful tools to evaluate the colloid chemistry in the precursor solution and observe the crystal nucleation and growth process of perovskite films [82]. Taking two FA-based spacers as examples (Fig. 1c), TTFA spacer with thieno[3,2-b]thiophene (TT) units result in stronger π-π interactions between spacer cations than BTFA spacer with 2,2′-bithiophene (BT) units. DLS measurement results (Fig. 1d) indicate that larger clusters were formed in TTFA-based precursor solutions with a negligible energy barrier (ΔG* or ΔG_0_*, Fig. 1e). *In situ* optical microscopy images (Fig. 1f) demonstrate that the larger clusters in TTFA-Pb films fostered the rapid growth of nuclei and the formation of larger crystals, which could improve the surface coverage and uniformity of TTFA-Pb films (Fig. 1g) [83]. In addition, *in situ* grazing incidence wide-angle X-ray scattering (GIWAXS) and *in situ* UV–vis absorption spectra can be employed to monitor the crystallization mechanism of RPP films, which can provide the key information on the phase composition and crystallization orientation [76, 83, 84]. The *in situ* GIWAXS results in Fig. 1h suggest that TTFA-Pb crystal nuclei required a lower formation temperature compared to the BTFA-Pb crystal nuclei and TTFA-Pb crystals preferred to grow perpendicular to the substrate. According to these characterization results, the proposed film formation process of TTFA-Pb and BTFA-Pb is illustrated in Fig. 1i [83]. Besides, the Fourier transform infrared spectra (FTIR), X-ray photoelectron spectra (XPS) and X-ray diffraction (XRD) patterns of different-stage films can be utilized to analyze the interactions between various components and the intermediates appearing during the film formation process.

### Managing Charge Transporting Ability

#### In-Plane Charge Transport

The charge transport in 2D RPPs shows the anisotropic characteristic, which mainly include two categorizes in a broad sense [85, 86]. The first type of charge transport refers to the charge transporting along the inorganic layer, which can also be termed as the in-plane charge transport. In this regard, if the crystal orientation of 2D RPPs is perpendicular to the substrate (out-of-plane growth direction), photogenerated charges can transport along the inorganic layer to charge transporting layers, which avoids the poor-conductivity organic layers and facilitates charge carrier separation and extraction in devices [87–89]. For high-n RPPs, the above-mentioned situation is easier to achieve as the crystal in high-n RPP films prefers growth in the vertical direction to substrate. By contrast, the low-n (n ≤ 5) RPPs suffer from difficulties in this aspect because the crystals in low-n RPP films are prone to grow along the in-plane direction and thus the bulky insulating organic spacers block charges to vertically transport to separation interfaces [53]. At this point, it is particularly vital for the low-n RPPs to manipulate their crystallization kinetics for realizing perpendicular crystallographic orientations. As mentioned in Sect. 2.1, the molecular structure of organic spacers plays an important role in this respect and the crystal growth direction can be managed via tuning the structure flexibility, size and chain length of spacer cations as well as selecting appropriate functional groups to functionalize spacer cations.

**Fig. 2:**
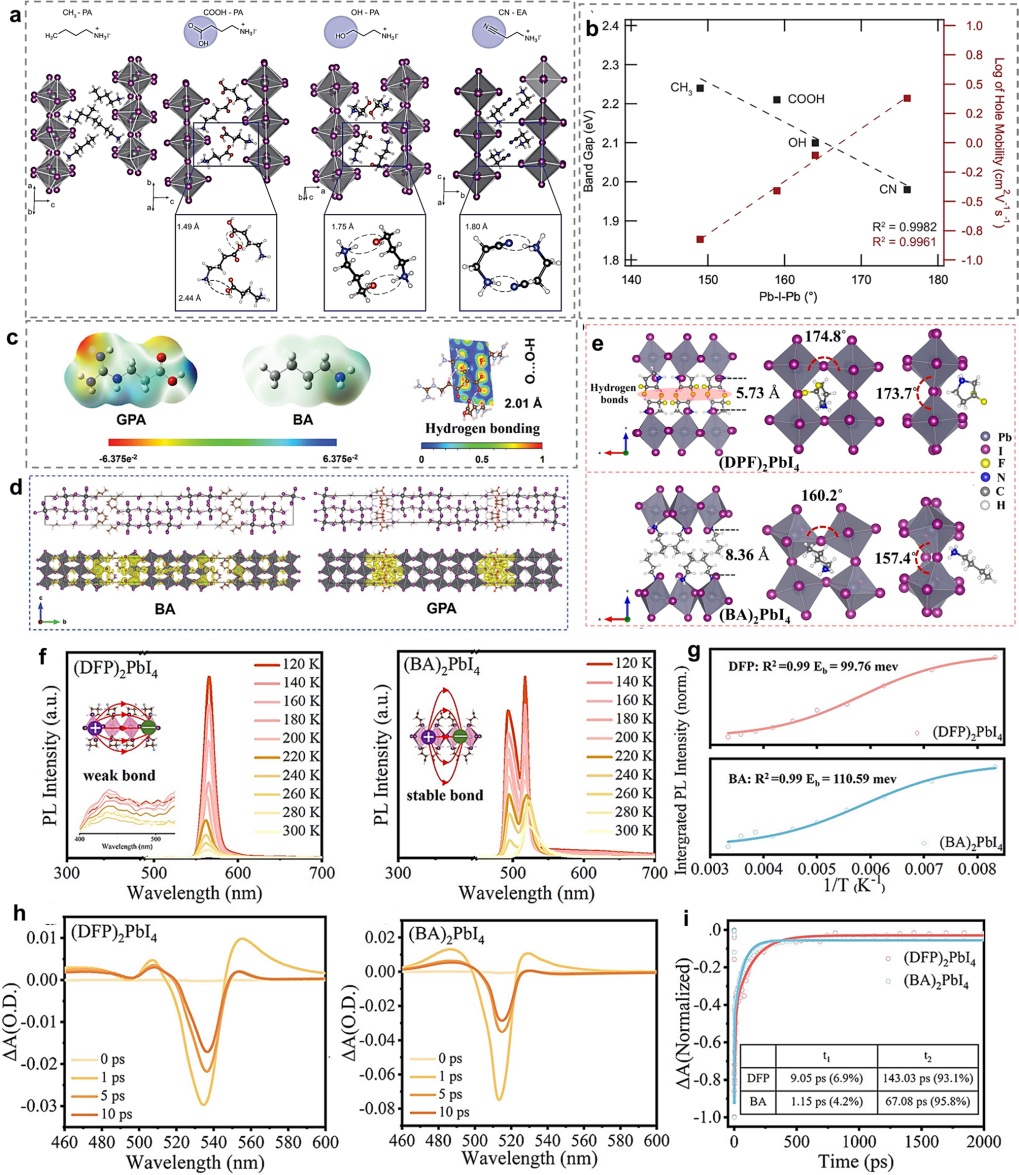
**a** Influence of different spacer cations on the distortion of [PbX_6_]^4−^ octahedra and **b** the corresponding relationship between the average Pb-X-Pb angle, *E*_*g*_ and hole mobility. Reproduced with permission [91]. Copyright 2022, Springer Nature. **c** Formation of HBs between GPA spacers. **d** Crystal structure and charge density of BA and GPA-based RPPs. Reproduced with permission [77]. Copyright 2024, Wiley–VCH GmbH. **e** Schematic diagram of (DFP)_2_PbI_4_ and (BA)_2_PbI_4_ single crystal structures showing the HB formation between DFP spacers. **f** Temperature-dependent PL spectra of (DFP)_2_PbI_4_ and (BA)_2_PbI_4_ RPP films. **g** Arrhenius diagram fits the curves of temperature-dependent PL spectra. **h** Femtosecond TA spectra at different time delays of (DFP)_2_PbI_4_ and (BA)_2_PbI_4_ RPP films. **i** Kinetic traces of the (DFP)_2_PbI_4_ and (BA)_2_PbI_4_ RPP films. Reproduced with permission [31]. Copyright 2024, American Chemical Society

In terms of the charge transporting ability along the inorganic layer, it is mainly determined by the structure of [PbX_6_]^4−^ octahedra. Generally, the distortion degree of [PbX_6_]^4−^ octahedra greatly affects the charge mobility in inorganic layers, which can be assessed by the equatorial and axial Pb-X-Pb angle. The less distortion of Pb-X-Pb angles (the average Pb-X-Pb angle is close to 180°) affords smaller bandgap and higher charge mobility in inorganic framework [90]. Organic spacers with different molecular structures can affect the distortion degree of [PbX_6_]^4−^ octahedra via different HB bonding modes with inorganic frameworks or different inter-spacer interaction modes, further influencing the charge transporting ability along the inorganic layer [91, 92]. Zhao and coworkers used different-structured organic spacers to tune the intermolecular interaction mode between adjacent organic layers and discovered the relationship between the average Pb-X-Pb angle, *E*_*g*_ and hole mobility (Fig. 2a, b) [91]. Note that distortion degree of [PbX_6_]^4−^ octahedra also affects the stability of RPP crystal structure, which will be discussed shortly.

#### Out-of-Plane Charge Transport

The second type of charge transport refers to the charge transporting between adjacent inorganic layers across organic spacer layers via the tunneling effect, which can also be regarded as the out-of-plane charge transport. Due to the insulating property and much smaller dielectric constant (*ϵ*_*r*_) of organic spacers than inorganic frameworks, organic spacers act as barriers and impede the charge transport [93]. In addition, the existence of VdW gaps between adjacent organic layers further increases the difficulty of charge transport between adjacent quantum wells (QWs) [94].

To improve the charge transporting ability between adjacent inorganic layers, the key is to decrease the barrier width and barrier height, both of which are closely bound up with organic spacers [95, 96]. The barrier width is mainly dependent on the size of spacer cations and thus appropriately decreasing the size of spacer cations is an effective way to reduce the tunneling distance [97–99]. The barrier height is related to the energy level alignment of inorganic layers and organic spacer layers. Increasing the intrinsic polarity and dipole moment of spacer cations can enhance *ϵ*_*r*_ and relieve the dielectric mismatch effect between organic spacers and inorganic layers, thereby promoting charge transport between adjacent inorganic layers [59, 100]. In addition, the nature conductivity of spacer cations affects the charge transfer between adjacent inorganic layers. As a rule, the higher conductivity of organic spacers can promote the out-of-plane charge transport, which can be achieved by designing organic spacers with π-conjugated systems [101–103]. Noteworthy that π-conjugated spacer cations may generate stronger orbital coupling interaction with Pb^2+^ and *p* orbital of I^−^ of inorganic framework, which increases the layer interaction and thus promotes charge transport [104, 105]. Moreover, introducing stronger intermolecular interactions between adjacent organic layers such as HB interactions and S–S interactions is capable of eliminating the energy gaps between adjacent organic layers and providing a bridge to dredge the charge transfer pathway [78, 80]. In this respect, the charge density and the electron localization function (ELF) can be calculated to investigate the intermolecular interaction role in the crystal electronic structure. As shown in Fig. 2c, d, the formation of HBs between neighbor GPA organic layers enables more charges to be localized in the interlayer region, which is favorable for interlayer charge transfer [77].

#### Characterization of Charge Properties

Multiple measurement and characterization methods can be performed to analyze the exciton dissociating and charge transport properties. The temperature-dependent PL spectra is a helpful tool to characterize the exciton property. *E*_b_ can be calculated by combining with integrated PL intensity and Arrhenius equation fitting [100]. Taking the recently reported 3,3-difluoropyrrolidinium (DFP) spacer as an example, DFP can generate HB interactions between two adjacent DFP spacer layers, which notably inhibits the octahedral lattice distortion and gives rise to a large Pb-I-Pb angle (over 170°, Fig. 2e). The temperature-dependent PL measuring and fitting results (Fig. 2f) indicate that DFP-based RPPs exhibit smaller *E*_b_ [31]. The smaller *E*_b_ value means the weaker Columbia force of electron–hole pairs, making them easier to dissociate to free carriers and laying the foundation of charge transport.

In addition, the ultrafast transient absorption (TA) measurement can be performed to further explore the exciton dissociation and free carrier generation processes as well as the charge carrier dynamics between different n-value phases in RPP films [16, 31, 106–108]. Generally, the broader photoinduced absorption and the lower photobleaching (PB) recovery are indicative of the increased and more efficient exciton dissociation. According to the TA spectra at different delay times, PB signals at different wavelength reflect the n-value composition in the RPP film [78]. As shown in Fig. 2h, the more obvious PB peak at 750 nm and the inconspicuous PB peaks between 500–700 nm suggest that the 3D-like phases (high-n value) are prominent while low-n value phases are suppressed in DFP-based RPP films. Through the kinetic traces and fits (Fig. 2i), the charge transfer between different n phases in DFP-RPP film is much faster, indicating that the charge transfer of DFP-based RPP film from 2D phases to 3D-like phases is more efficient than that in BA-RPP film [31].

Furthermore, the charge transporting property at interface in solar cells can be evaluated by the time-resolved photoluminescence (TRPL), transient photovoltage (TPV) decay, transient photocurrent (TPC) decay and electrochemical impedance spectroscopy (EIS) measurements. For TPV and TPC delay measurements, the longer photovoltage decay life and shorter photocurrent decay time manifest the extended charge carrier lifetime, expediated charge extraction and collection efficiency, and inhibited nonradiative recombination [59, 109]. For EIS measurements, the larger recombination resistance (*R*_rec_) and the smaller charge transfer resistance (*R*_ct_) fitted from the EIS data demonstrate the suppressed charge recombination and more efficient charge transport [57, 104].

### Affecting Stability of RPPs

#### Hydrophobicity

It is quite obvious that the superior moisture stability of 2D RPPs is related to the hydrophobicity of bulky organic spacers. The non-ammonium terminus of organic spacers with high hydrophobicity can function as good barriers to preserve the crystal structure from the invasion of water. With this in mind, changing the hydrophobicity of bulky organic spacer cations can adjust the moisture resistance of RPPs. In this respect, the large-sized, long-chain, fluorinated and strong π-conjugated bulky spacer cations can theoretically enhance the stability of RPPs against water because of their stronger hydrophobicity [110, 111]. The hydrophobicity of RPP films can be evaluated by water contact angle measurement. The surface contact angles (CAs) with water droplets of benzylamine (BZA), DF-BZA-based quasi-2D RPP films and 3D FAPbI_3_ perovskites are 50.83°, 66.19°, and 43.60°, respectively [56]. Apparently, the DF-BZA spacer cation with more hydrophobic fluorinated non-ammonium terminus led to larger CAs, meaning the stronger hydrophobicity of the perovskite films. Noteworthy that the hydrophobicity of spacer cations affects the moisture stability of RPPs but is not the most critical intrinsic factor to guarantee the excellent stability of RPPs. The influence of organic spacers on the stability of RPP films should be elucidated from a more in-depth level.

#### Lattice Stability

**Fig. 3:**
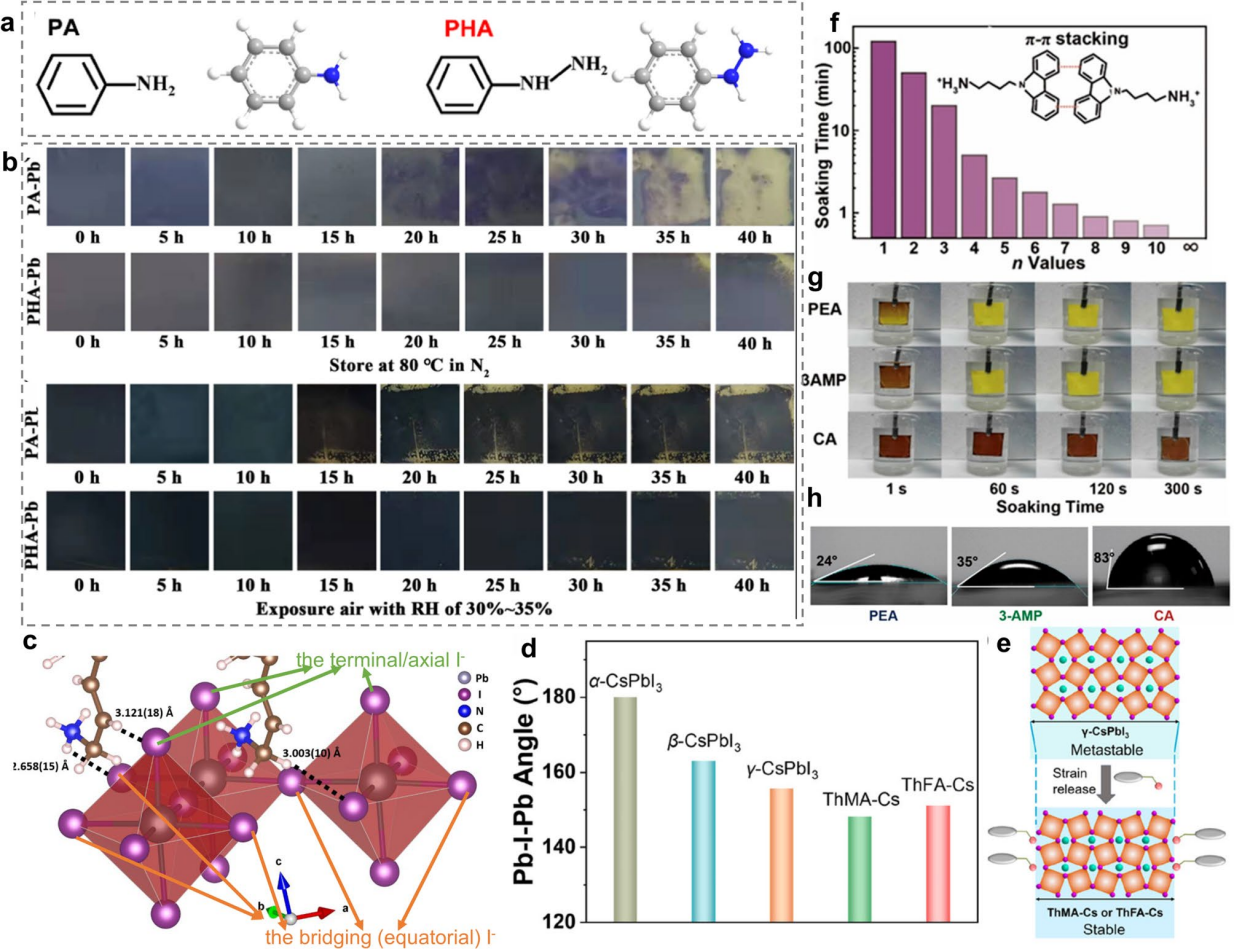
**a** Chemical structure of PA and PHA spacers. **b** Influence of PA and PHA spacers on RPP film stability at different conditions. Reproduced with permission [113]. Copyright 2022, American Chemical Society. **c** Three types HBs between PPA spacer and inorganic framework. Reproduced with permission [115]. Copyright 2020, American Chemical Society. **d** Average Pb-I-Pb angle for different-phase CsPbI_3_ perovskites and ThMA, ThFA-based CsPbI_3_ RPPs. **e** Schematic diagram showing the strain release of γ-phase CsPbI_3_ with ThMA and ThFA spacers. Reproduced with permission [117]. Copyright 2020, American Chemical Society. **f** Schematic illustration of π-π stacking interactions between CA spacers. **g** Considerably enhanced water resistance of CA-based RPPs. **h** CAs of CA-based RPP film compared with other counterparts (3AMP = 3-(aminomethyl)piperidine, a spacer for DJ perovskite). Reproduced with permission [101]. Copyright 2022, American Chemical Society

In essence, the stability of RPPs is intrinsically determined by the lattice stability, which depends a lot on the spacer–inorganic framework and inter-spacer interactions. In general, the stronger HB interaction between organic spacers and inorganic frameworks can help to stabilize the RPP assembly [112]. Thus, designing spacer cations to generate stronger HB interaction with inorganic layers is a feasible way to enhance the stability of RPPs. For example, in comparison with phenylammonium (PA) spacer (Fig. 3a), PHA spacer with one more -NH- group at the ammonium terminus connect [PbI_6_]^4−^ octahedra by stronger N–H…I HB interactions. Under the extreme environments, it can be observed that PHA-based RPP films experienced a slower color change than PA-based RPP films, suggesting the better heat and humid stability for PHA-based RPPs than PA-based RPP films (Fig. 3b) [113].

From another perspective, the structure distortion degree of the inorganic octahedra framework affects the stability of RPP lattices. The larger average Pb-I-Pb angle indicates the smaller structure distortion and more stable crystal structure. By contrast, the smaller average Pb-I-Pb angle means the more severe structural deformation, which harms the stability of RPP lattices. The structure distortion degree can be tuned by adjusting the HB bonding mode between spacer cations and inorganic layers. To be specific, the types of I^−^ in inorganic frameworks to form HBs with organic spacers include two categories, namely the bridging I^−^ and the terminal I^−^, which are also referred as the bridging equatorial I^−^ and the axial I^−^ in some reports [114, 115]. The bridging (equatorial) I^−^ is located at the second nearest neighbored atomic layer, while the terminal/axial I^−^ is coordinated by only one Pb^2+^ [114]. Different molecular structures of organic spacers may enable them to form HBs with different I^−^, resulting in the different degrees of structure deformation and further influencing the stability of crystal structure. Xi et al. designed the 3-phenyl-2-propenammonium (PPA) spacer and found three types of HBs, including − NH···I (the bridging equatorial I^−^), − CH_2_···I (the bridging equatorial I^−^) and − CH···I (axial I^−^) (Fig. 3c) [115]. Such HB bonding modes gave rise to large equatorial and axial Pb-I-Pb angle of 179.9° and 176.4°, resulting in less distortion of inorganic octahedra and enhancing the lattice stability [115].

Noteworthy that the distortion degree of inorganic framework in CsPbI_3_-based RPPs is closely related to the phase stability of CsPbI_3_. It has been reported that the average Pb-I-Pb angle in CsPbI_3_ perovskite reduces from 180° for the α-phase to 163° for the β-phase, 155° for the γ-phase, and 95° for the δ-phase [116]. Based on this, designing organic spacers with different molecular structures can tune the distortion degree of inorganic framework of inorganic framework to stabilize CsPbI_3_ at a certain phase. The 2-thiophenemethylamine hydroiodide (ThMA) and 2-thiopheneformamidine hydroiodide (ThFA) spacer led to the average Pb-I-Pb 148° and 151° [117], which can stabilize the CsPbI_3_ perovskite at γ-phase (Fig. 3d). The slightly smaller average Pb-I-Pb angle than that of the γ-CsPbI_3_ (155°) means that the inner stress can be released after the CsPbI_3_ being tailored with ThMA and ThFA spacers (Fig. 3e) [117].

Besides, the stability of RPP crystal structure can be intensified by enhancing the inter-spacer interactions. The introduction of HB interactions, strong π-π interactions as well as S–S interactions between adjacent organic layers can assist in stabilizing the crystal structure of RPPs against adverse environmental factors like humidity and heat [118]. Compared with BA spacer cations, HB interactions between GPA spacers induced a lower formation energy for (GPA)_2_(MA)_4_Pb_5_I_16_, indicating the better stability of GPA perovskite framework [77]. In another case, compared with BA, the degradation of RPP films to produce PbI_2_ under heat and humid stress was inhibited when using MTEA spacers because the S–S interactions between MTEA spacers offer the stabilization of RPP framework against heat and humidity [80]. Additionally, benefitting from the strong π-π stacking interactions between (9H-carbazol-9-yl)butyl-1-ammonium (CA) spacers, CA-based RPP films exhibit excellent resistance to water. It is surprising to find that the CA-based RPP films with n = 5 presented a negligible change in water after 300 s (Fig. 3f, g). The surface CAs of the CA-based RPP film reached 83° (Fig. 3h), mirroring the superior hydrophobicity of CA-based RPPs films [101].

#### Defects

Moreover, some other factors such as defect density, morphology and crystallinity influence the stability of RPP films, all of which can be adjusted via organic spacers. Defects have been recognized as an origin of the degradation of perovskite films and their existence seriously jeopardizes the RPR film stability [119]. According to previous reports, defects are more likely to appear at grain boundaries, which are vulnerable to various environmental factors [120]. In this regard, it is of great importance to design spacer cations to prepare large-grain and high-crystallinity RPP films with decreasing grain boundaries, which play a crucial role in preventing the invasion of adverse factors [79, 121]. Besides, spacer cations themselves can act as defect passivators in RPP films. Spacer cations featured with an electron-donating moiety can interact with uncoordinated Pb^2+^ to passivate undercoordinated lead defects and thus enhance the film stability [79, 122]. Zhen et al. proposed that a small amount of Gly spacer can stay at the crystal boundary to fill the trap sites. The O atoms of Gly can also fill the oxygen vacancy generated under UV light, thus improving the stability against UV light [79].

#### Characterization of Stability

In terms of the characterization methods of the RPP stability, in addition to the above-mentioned water contact angle measurement, XRD, UV–vis absorption spectra and SEM images of RPP films aging under different conditions have been employed to assess the RPP film stability [57]. In XRD patterns, the appearance degree of PbI_2_ peaks combining with the fading degree of aging RPP films can reflect the degradation rate of the RPP film stability [57, 117]. The device stability is often evaluated via the variation degree or remaining percentage of initial photovoltaic performance parameters at various aging conditions including continuous light illumination, high-temperature and highly-humid conditions. In particular, maximum power point tracking (MPPT) under at different conditions has become an authoritative and widely used method to confirm the device operation stability [59, 78, 123, 124].

## Rationally Designing Organic Spacers for High-Performance RP PSCs

### Design Considerations of Organic Spacers

#### Steric Configuration

The optoelectronic properties and stability of RPP can be manipulated by the steric configuration of spacer cations, which includes the adjustment of space size/chain length, the construction of symmetric and asymmetric structure, the selection of functional group location and the design of shape of spacer cations [54, 125–129]. Surprisingly, the small changes of the steric configuration of spacer cations may invoke huge changes of structure and properties of RPPs [130]. In this scenario, it is meaningful to take these steric configuration aspects into full consideration to elaborately design spacer cations for creating RPPs with attractive stability and optoelectronic properties.

**Fig. 4:**
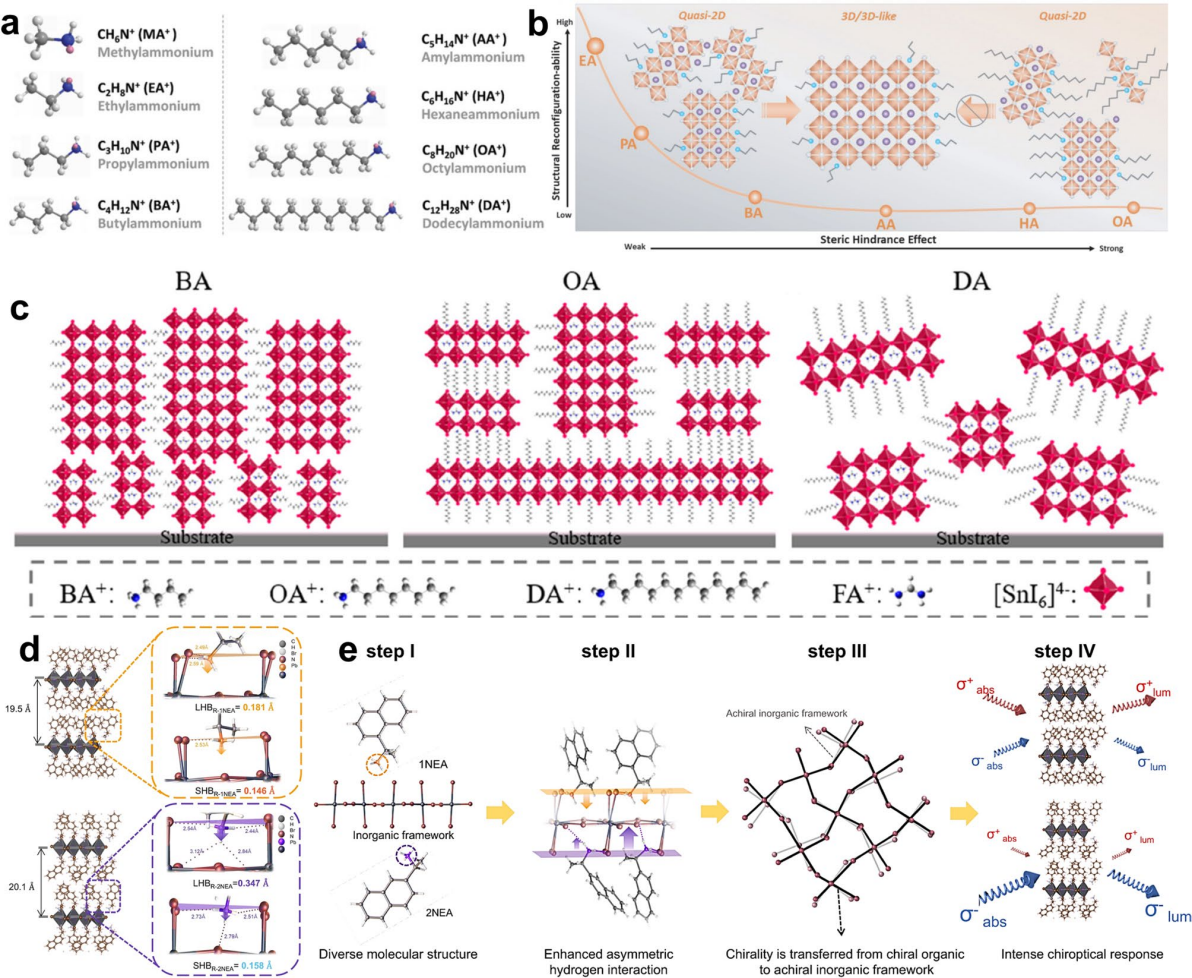
**a** Chemical structure of alkylammonium spacers with different chain length. **b** Schematic illustration of the influence of steric hindrance of alkylammonium spacers with different chain length on thermally assisted structural reconfiguration of RPPs. Reproduced with permission [132]. Copyright 2022, Wiley–VCH GmbH. **c** Schematic illustration of the influence of chain length of organic spacers on crystal orientation, dimensionality, and phases distribution of A’_2_(FA)_n-1_Sn_n_I_3n+1_. Reproduced with permission [133]. Copyright 2020, American Chemical Society. **d** Schematic diagram showing the asymmetric HB interaction between chiral spacer cations and inorganic frameworks and the influence of functional group location on the HB interaction mode. **e** Schematic diagram illustrating the chirality transfer process from chiral spacer cations to chiral RPPs. Reproduced with permission [138]. Copyright 2023, Springer Nature

Basically, the formation of 2D RPP crystal structure relies too heavily on the size of organic ammonium cations. If the size (ionic radius) of organic ammonium cations is small, making the tolerance factor (t, t = (r_A_ + r_X_)/√2 (r_B_ + r_X_)) within 0.8–1, these cations will fit into the 3D [PbX_6_]^4−^ frameworks, stay in the A-site and remain 3D perovskite crystal structure. Only when the ionic radius of spacer cations is large to make t > 1, can these cations separate the 3D perovskite architecture into layers and form 2D structures [131]. Additionally, different size and steric structure of spacer cations bring different steric hindrance effect, which has been found to affect the phase distribution, redistribution, moisture and thermal stability of RPPs [125]. For instance, Zhang and co-workers have found that the alkyl ammonium spacer cations with longer chain length yielded larger steric hindrance between adjacent perovskite domains, which not only rendered a more hydrophobic surface of the resulting RPP films but also suppressed the increased-dimensional phase redistribution during a thermal aging process (Fig. 4a, b) [132]. In spite of the enhanced thermal stability and hydrophobicity, too long chain length of spacers may have a negative effect on the crystal orientation and phase distribution of RPP films for their photovoltaic applications. For example, the employment of long-chain alkylammonium-like octylamine (OA) and dodecylamine (DA) to prepare 2D Sn-based RPPs can cause unsatisfactory crystal orientation and phase distribution compared with BA spacer (Fig. 4c) [133]. Thus, the size and chain length of organic spacers should be elaborately regulated to tune the film quality and stability by considering the above aspects in an integrated manner.

Furthermore, the steric configuration of spacer cations determines their interaction mode with inorganic frameworks with a large degree, which further affects the crystal structure of inorganic frameworks and results in different optoelectronic and physiochemical properties of RPPs. A typical example is that organic spacers with inherent chirality will confer RPPs with a chiral crystal structure and unique properties for various potential applications [134–137]. It has been uncovered that the chirality transfer from organic spacers to 2D RPPs is realized via the asymmetric HB interaction between chiral spacer cations and inorganic frameworks. Furthermore, the functional group location plays a vital role in the chirality transfer process as different functional group locations lead to different asymmetric HB interactions (Fig. 4d) [138]. In recent years, chiral RPPs have become an emerging area of hot research and their unique chiral crystal structure and spin-related properties may bring in some potential benefits for the efficiency and stability of solar cells. At current stage, many 2D chiral RPPs have been prepared and used for photodetectors, while their application in solar cells is very limited. In 2022, Lioz Etga and coworkers integrated chiral quasi-2D RPPs (ABX_3_ χ (S/R-MBA)_2_PbI_4_) into solar cells for the first time [139]. It is discovered that at high χ values, the chirality is more pronounced and affects the current density of solar cells more than at low χ values [139]. In 2023, Lucas Scalon et al. also realized the application potentials of chiral perovskites into photovoltaic technology [140]. Overall, the chiral RPPs derived from the inherent chirality of spacer cation bring in new hope and likelihood for the development of solar cells. Therefore, more attempts are encouraged to uncover the correlation between the chiral crystal structure of RPPs and the film quality, charge transporting property and photovoltaic performance of solar cells in future study.

#### Dipole Moment

**Fig. 5:**
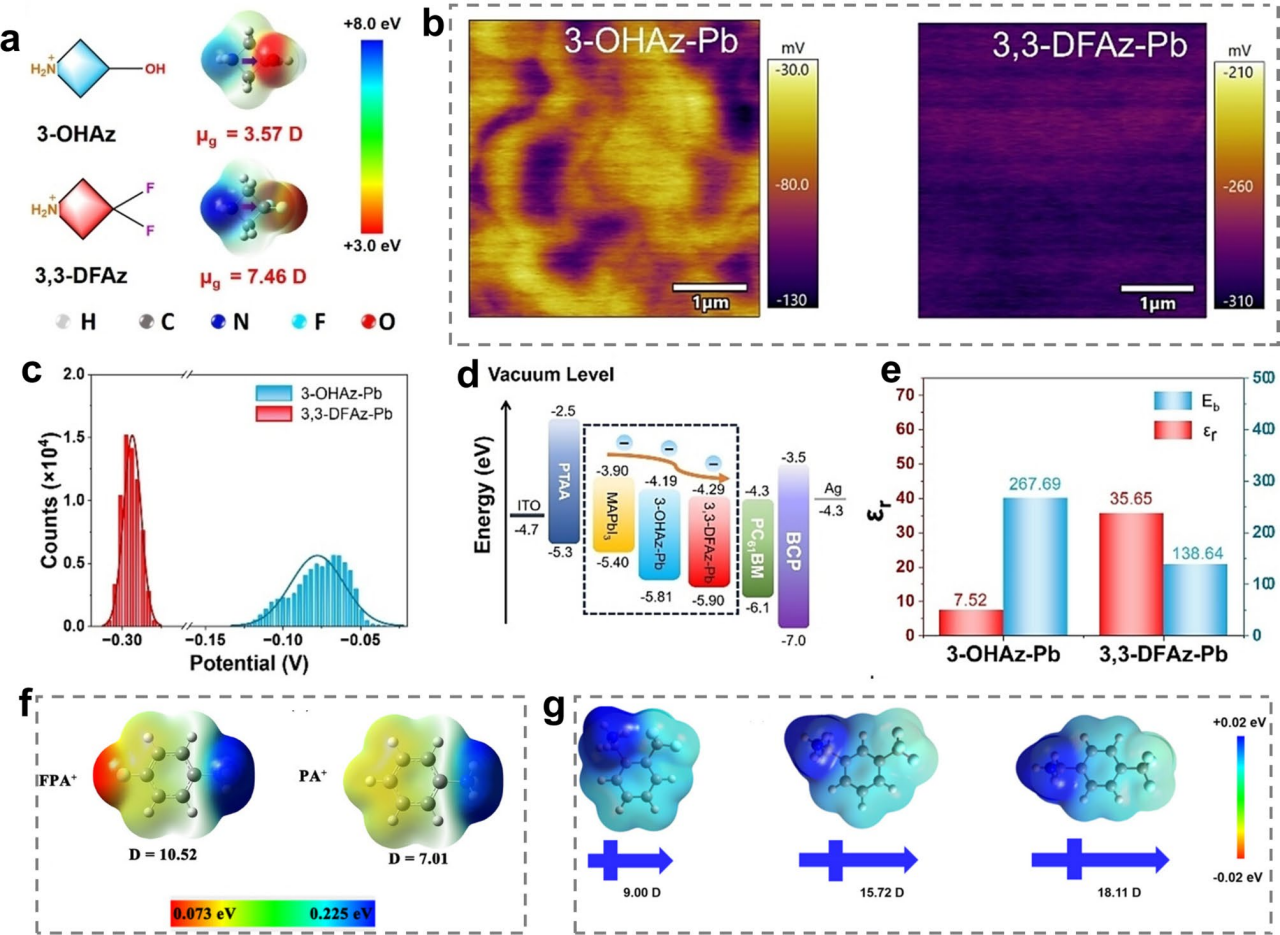
**a** Chemical structure of 3-OHAZ and 3,3-DFAz spacers with different dipole moments. **b** 2D surface potential images, **c** CPD histograms, **d** energy level alignment and **e** *E*_*b*_ and *ε*_*r*_ of 3-OHAZ and 3,3-DFAz-based RPPs. Reproduced with permission [58]. Copyright 2024, Wiley–VCH GmbH. **f** Influence of F substitution on dipole moments of organic spacers. Reproduced with permission [148]. Copyright 2022, Wiley–VCH GmbH. **g** Influence of different -CF_3_ group substitution position on dipole moments of organic spacers. Reproduced with permission [147]. Copyright 2024, Wiley–VCH GmbH

The dipole moment can reflect the polarity and charge distribution uniformity of organic spacers, which determines *E*_*b*_. Generally, the larger dipole moment of organic spacers represents the higher polarity and larger *ϵ*_*r*_ of organic spacer, which can decrease the dielectric mismatch between inorganic layers and organic layers, thereby resulting in smaller *E*_*b*_ and promoting electron–hole separation [54, 141, 142]. Also, the dipole moment of organic spacers can affect the energy band structure and the Fermi level (*E*_*f*_*)* of RPPs [143]. Some researchers achieved the flexible control of *E*_*b*_ and energy band structure of RPPs via modulating dipole moment of organic spacers [58]. Compared with 3-hydroxyazatidine (3-OHAz) spacer, the 3,3-DFAz spacer with two strongly electronegative -F groups show larger dipole moment (Fig. 5a). The kelvin probe force microscopy (KPFM) characterization (Fig. 5b) and the quantitative analysis of the contact potential difference (CPD) histogram (Fig. 5c) demonstrate that 3,3-DFAz spacer conferred RPP films with lower surface potential and narrower potential dispersion. The lower average CPD of 3,3-DFAz-based RPP films indicates the *E*_*f*_ closer to the conduction band (CB). In addition, the shifts of energy bands and work function was investigated by the ultraviolet photoelectron spectrometry (UPS). The work function, valence band maximum (VBM), and CB minimum (CBM) of 3,3-DFAz-based RPP films all exhibited a larger shift from the vacuum level compared to 3-OHAz-based RPP films. As shown in Fig. 5d, the CBM of 3,3-DFAz-based RPP film is lower and closer to that of electron transporting layer (ETL). Both the improved *E*_*f*_ and enhanced energy level can promote the charge transfer and collection. Furthermore, *E*_*b*_ and *ϵ*_*r*_ were calculated by fitting the temperature-dependent PL spectra and testing the capacitance frequency curve. The obtained *E*_*b*_ and *ϵ*_*r*_ are summarized in Fig. 5e. It is clear to see that 3,3-DFAz spacer with higher dipole moment resulted in lower *E*_*b*_ and higher *ϵ*_*r*_ of RPP films, which can promote the separation of excitons into free carriers and suppress their recombination [58].

In light of the significance of organic spacers’ dipole moment on exciton and charge properties of RPPs, it is imperative to take this point into account when designing organic spacers. The dipole moment of organic spacers links with their composition, structure, size, etc. [104, 144–146]. The chemical composition of organic spacers has a notable influence on the dipole moment. Compared with organic spacers composed of only C, N, and H elements, organic spacers containing halogen and chalcogen elements are characteristic of larger dipole moment [141, 147]. For instance, the 4-fluorobenzenaminium (FPA) spacer with -F group exhibits a larger dipole moment than the unfluorinated benzenaminium (PA) spacer (Fig. 5f) [148]. Such a result may be ascribed to the high electronegativity of halogen and chalcogen elements, which can strongly pull electrons and thus lead to high dipole moment [128, 145]. This also provides a rational explanation why halogen and chalcogen elements-based functional groups have been widely used to modify organic spacers for RPPs [142, 149]. Besides, it is worth noting that the appearing position of these functional groups on organic spacers also affects the dipole moment because of the changed charge distribution [147, 150]. As illustrated in Fig. 5g, adjusting the substitution position of -CF_3_ group on the benzene ring from the ortho position to the meta and para position increases the dipole moment of organic spacers [147].

#### Intermolecular Interactions Between Organic Spacers

As discussed in Sect. 2, intermolecular interactions between organic spacers play a significant role in modulating the crystallization process, affecting the charge transport property and tuning the stability of RPPs. Thus, it is necessary to consider the inter-spacer interactions when designing organic spacers. The original weak VdW force between adjacent organic layers brings about relatively large VdW gap, which is undesirable for charge transport and stability of RPPs. To overcome this weakness, designing spacer cations to form stronger intermolecular interactions is an implemental way, which should particularly focus on the design of non-ammonium terminus by the control of the chain length and the selection of functional groups [71, 151]. Increasing the chain length of alkyl spacer cations not only renders stronger hydrophobicity of organic spacers but also enhances the VdW interaction between organic spacers, which further participates in adjusting the crystallization process of RPPs [71]. In addition, modifying the non-ammonium terminus via specific groups provides great opportunities to form other non-covalent interactions between adjacent organic layers, such as HB, halogen bonding (XB), π-π, S–S interactions, holding the potential to optimize film formation process, promote charge transport and increase crystal structure stability of RPPs [78, 80, 100, 152].

Honestly, the intermolecular non-covalent interactions between organic spacers are more valuable than what we mentioned above [153]. An extraordinary significance of strong intermolecular interactions between spacer cations has been expounded by Najarian and co-workers [154]. The strong homomeric non-covalent interactions can lead to the formation of organic scaffold sublattices, which offer a new avenue for templating hybrid lattices with controlled distortion and orbital arrangement and overcoming restrictions in conventional perovskites. They introduced homomeric non-covalent bonds (HBs and XBs) between spacer cations by designing organic spacers with self-complementary properties, which can interlock with an inorganic framework and influence the geometric arrangement and electronic configuration of the crystal. Unprecedentedly, this principle of design overcomes the geometrically and energetically advantageous distortion and enables octahedral perovskites beyond what is postulated in Goldschmidt’s rule. In consequence, some novel octahedral perovskites like Ge and Cu-based perovskites with excellent optoelectronic properties can be obtained, showing great potential for photovoltaic applications [154, 155].

### Molecular Structure Design Strategies of Organic Spacers for High-Performance RP PSCs

**Fig. 6:**
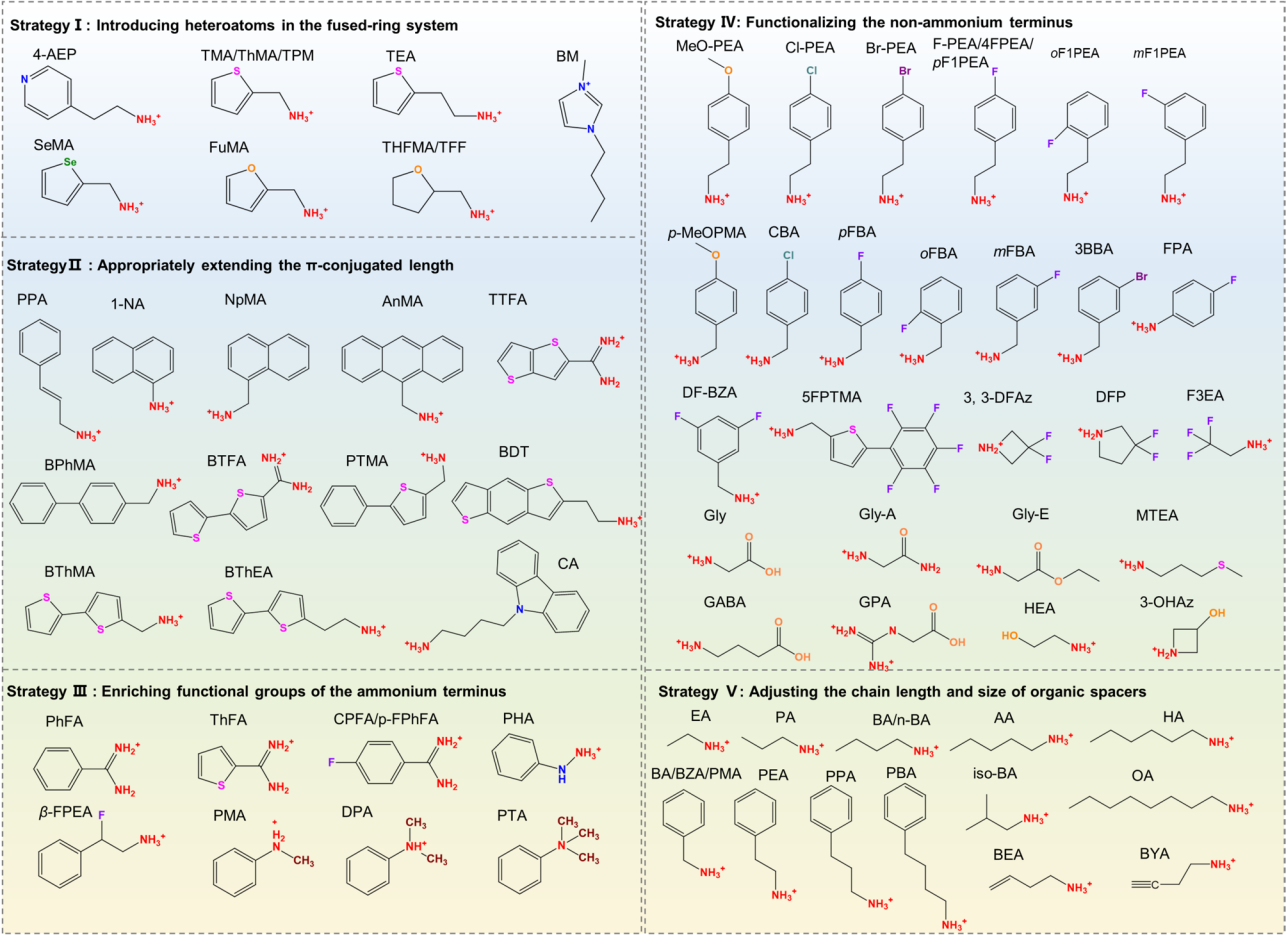
Proposed molecular structure design strategies for organic spacers and the chemical structure of organic spacers designed according to these strategies

In the early stage of research on RP PSCs, researchers took n-BA, PEA and PMA spacers-based RPPs as the major study object. Afterward, along with the discovery of the vital influences of organic spacers on the photovoltaic performance of RP PSCs, an increasing number of research have been carried out to design novel organic spacers for enhanced RP PSCs. To clarify the design principles of organic spaces for enhancing photovoltaic performance of RP PSCs, this section proposes some useful molecular structure design strategies of spacer cations. The molecular structure of organic spacers designed by these strategies is summarized in Fig. 6.

#### Strategy I: Introducing Heteroatoms in the Fused Ring System

Compared with pure-carbon fused rings like benzene rings and cyclohexane rings, fused rings containing heteroatoms such as N, S, O, and Se afford organic spacers more opportunities to tune the optoelectronic properties and stability of RPPs, which can be reflected in three aspects. Firstly, the appearance of heteroatoms with different electronegativity and atomic radius can adjust the polarity, dipole moments and dielectric property of organic spacers, which further influence *E*_*b*_ and charge transport properties of RPPs. Secondly, these heteroatoms provide possibilities to strengthen the inter-spacer interactions, which can promote charge transport and stabilize the crystal structure [142]. Moreover, the existence of heteroatoms can interact with inorganic components, like Pb^2+^ in precursor solutions and in inorganic frameworks of the final RPP film, which plays an important role in modulating the crystallization process and passivating defects [122, 156]. In recent years, a variety of heteroaromatic ring (such as pyridine, thiophene, selenophene, and furan)-based organic ammonium cations including 4-AEP [81], ThMA [121], ThFA [157], 2-thiopheneethylammonium (TEA) [149, 158–160], 2-selenophenemethylammonium (SeMA) [142], and 2-furfurylammonium (FuMA) [161] have been designed as spacers for RPPs, which represent promising candidates for highly-efficient and stable PSCs.

**Fig. 7:**
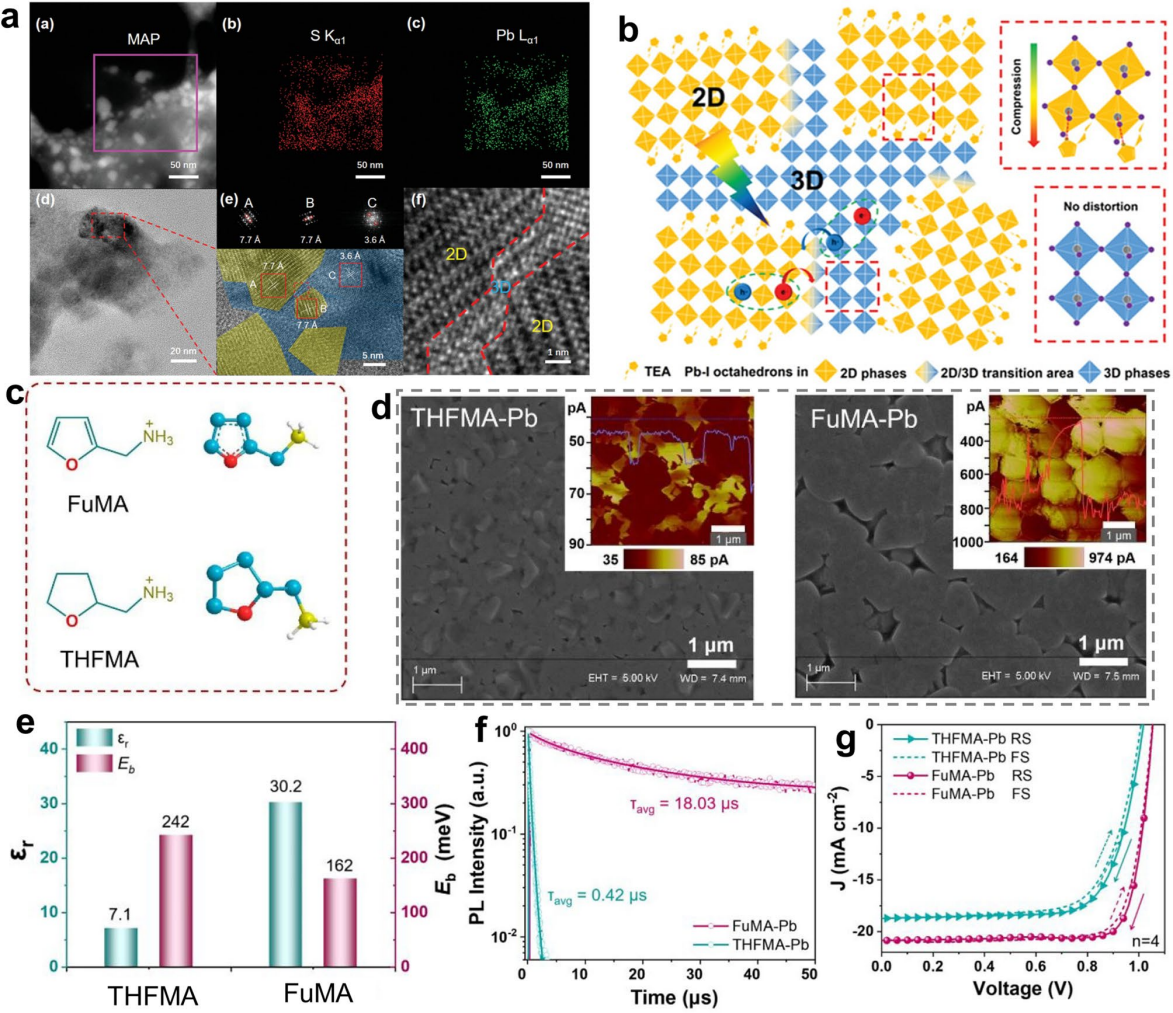
**a** STEM images and corresponding EDS elemental mapping as well as TEM and magnified HR-TEM images of TEA-based RPPs. **b** Schematic diagram showing the 2D/3D bulk heterojunction structures. Reproduced with permission [149]. Copyright 2019, WILEY–VCH Verlag GmbH & Co. KGaA, Weinheim. **c** Chemical structure of FuMA and THFMA spacer. **d** SEM images of FuMA and THFMA-based RPP films with c-AFM images inside. **e** *E*_*b*_ and *ε*_*r*_, **f** PL decay curves of FuMA and THFMA-based RPP films. **g** *J*-*V* curves of FuMA and THFMA-based RP PSCs. Reproduced with permission [164]. Copyright 2022, American Chemical Society

As mentioned in Sect. 2.1, the 4-AEP spacer can slow down the crystallization rate of perovskite via the coordination interaction between N atoms of pyridine ring and Pb^2+^, which yielded high-quality RPP films with full coverage, smooth surface, and uniformly distributed grains. As a result, (4-AEP)_2_MA_4_Pb_5_I_16_ (Fig. 7b) RP PSCs delivered a PCE of 11.68% with good air stability, which was much better than PEA_2_MA_4_Pb_5_I_16_-based devices [81]. In another study, the strong interaction between the heteroatom (S) of thiophene ring and Pb^2+^ drove the compression-induced epitaxial growth of the 3D phase at the grain boundaries of the 2D phase, leading to the 2D/3D bulk heterojunction structures with 3D perovskite phases embedded in 2D perovskite matrix (Fig. 7a, b). Such structures rendered TEA-based RPPs with longer exciton diffusion length, extended charge carrier lifetime and better stability than PEA-based RPPs. In consequence, TEA_2_MA_3_Pb_4_I_13_ RP PSCs achieved a higher PCE (7.22%) than PEA_2_MA_3_Pb_4_I_13_ RP PSCs (4.69%). Further by introducing NH_4_Cl additives and optimizing the solvent, the PCE for TEA_2_MA_3_Pb_4_I_13_ RP PSCs was improved to 11.32% with negligible hysteresis and impressive ambient stability [149]. Subsequently, MA-free (TEA)_2_(FA)_n‑1_Pb_n_I_3n+1_ RPPs were studied and the devices yielded a higher PCE of 21.0% for n = 10 RPPs and 18.16% for n = 5 RPPs [23]. The better performance in this case compared with that in the last case may be mainly ascribed to the optimized spatial phase distribution by varying the sequence of incorporating TEAI, which contributed to the lower energy disorder and more effective charge transport. The two studies imply that the structure tailoring of organic spacer is the foundation for high-performance RP PSCs and then the device performance can be further improved by optimizing the preparation technique to tightly control the film quality.

Compared with the thiophene unit, the furan unit with the O atom which possesses smaller atomic radius and larger electronegativity can generate the enhanced intermolecular orbital overlap and carrier transport in 2D RPPs [162]. FuMA-based RP PSCs achieved a PCE of 15.66% with the assistance of methylammonium chloride (MACl). Nevertheless, in terms of intermolecular interactions, selenophen-based spacer cations are able to form stronger intermolecular Se-Se interaction because of the extensive π-overlap of the *p*-orbitals of Se atoms, which can improve the charge transporting ability [23, 163]. (SeMA)_2_MA_4_Pb_5_I_16_ and (SeMA)_2_FA_4_Pb_5_I_16_ RP PSCs delivered a high PCE of 13.59% and 15.57%, respectively. Further through interface engineering, the PCE can be boosted to 17.25% and 19.03%, respectively. These RP PSCs also exhibited decent stability against heat, humidity and light soaking [142].

In addition to various heteroaromatic rings, several aliphatic heterocyclic rings such as the epoxybutane ring and piperidine ring have been utilized for spacer design of RPPs. In comparison with heteroaromatic rings, aliphatic heterocyclic rings are featured with better structure flexibility but the absence of π-conjugated structure, which results in different physiochemical properties. Wang et al. compared two types of O-bearing fused ring-based spacers for RPPs, namely FuMA and (tetrahydrofuran-2-yl) methanaminium (THFMA, Fig. 7c). Compared with THFMA spacer, FuMA spacer with a conjugated furan unit conferred RPPs with better film conductivity (Fig. 7d), larger *ϵ*_*r*_ and smaller *E*_*b*_ (Fig. 7e) as well as an ultralong average carrier lifetime (Fig. 7f). Consequently, FuMA-based 2D RP PSCs achieved a high PCE of 18.0% with better durability (13.79% for THFMA-based RP PSCs, Fig. 7g) [164]. In another case, TFMA (TFF) spacer was utilized to collaborate with TMA (TPA) spacer for binary spacer cation RPPs. The introduction of THFMA spacer led to the slight structural distortion of the equatorial Pb-I-Pb bond angles, extended emission lifetime of RPPs and improved photovoltaic performance of RP PSCs [165]. According to the aforementioned studies, we speculate that heteroaromatic ring-based organic ammonium cations exhibit greater advantages in serving as unary organic spacers than aliphatic heterocyclic ring-based analogues. This possible reason may be that the presence of π-conjugated moiety can bring strong π-π interactions between spacer cations and strong π-Pb interactions with inorganic layers, which play a vital role in optimizing the film quality and optoelectronic properties of RPPs. By contrast, aliphatic heterocyclic ring-based organic ammonium cations with better structure flexibility are more suitable to act as a supplementary/second organic spacer to adjust the crystal structure and properties of RPPs.

As for this design strategy, it should note that the N atoms at the fused rings like pyridine ring, pyrrole ring, imidazolium ring and piperidine ring are easily protonated during the synthesized process, making these fused ring-based organic ammonium become bivalent spacer cations, which is applicable for Dion–Jacobson (DJ) perovskites rather than RPPs [166, 167–170, 171]. Luckily, this characteristic can make imidazolium ionic liquids as spacers for RPPs, in which the ammonium terminus is not necessary and the HB can form between C_2_-H of imidazolium ring and I^−^ of inorganic frameworks. According to this criterion, 1-butyl-3-methylimidazolium (BM) has been designed as spacer to be used in composition with BA. The formed (BM)_2-x_(BA)_x_MA_n-1_Pb_n_I_3n+1_ RPPs-based devices delivered an impressive PCE of 14.2% and 17.3% for n = 3 and n = 4 RPPs [172].

#### Strategy II: Appropriately Extending the π-Conjugated Length

For π-conjugated spacer cations, extending the π-conjugated length provides a helpful way to manipulate *E*_*b*_, charge transport capability and stability of RPPs. On the one hand, extending the π-conjugated length can increase the dipole moment and *ϵ*_*r*_ of organic spacers, thereby reducing the dielectric mismatch between organic layers and inorganic layers. Accordingly, the *E*_*b*_ of 2D RPPs can be reduced and the charge carrier separation can be promoted [104]. On the other hand, the longer π-conjugated length can give rise to stronger π-π interactions between organic spacers, which afford more rigid organic layers and enhance the lattice stability [101]. From the perspective of hydrophobicity and steric hindrance effect, extending the π-conjugated length can increase the hydrophobicity and steric hindrance effect of organic spacers, which play a critical role in increasing the water resistance and inhibiting the ion migration. Furthermore, it appears that organic spacers with larger π-conjugated size are more likely to contribute the π-electrons to the CBM which is mainly dominated by the* p*-orbitals of Pb^2+^, consequently reducing *E*_*g*_ and promoting the absorption of photons to generate more electron–hole pairs. More importantly, the π and π* states located in the near-edge CB may improve the electronic coupling between organic layers and inorganic layers, which is in favor of the charge mobility and photovoltaic performance [173].

According to previous literatures, extending the π-conjugated length can be implemented mainly by three ways. The first way is to introduce a carbon–carbon double bond (-C = C-) into aromatic spacer cations and connect the aromatic ring by a carbon–carbon (-C–C-) single bond. The PPA spacer is designed by this way. The insertion of -C = C- bond with *sp*^2^ hybridization allowed the spacer to form an extra HB bond (-CH_2_···I) with the inorganic framework, making PPA spacer protrude much deeper into the grooves formed by the terminal I^−^ of the [PbI_6_]^4−^ octahedra. Such an unusual structure feature results in less distortion of RPP crystal structure and enhances the structure stability. PPA-based RP (n = 4) PSCs achieved a PCE of 14.76%, which can remain 93.8 ± 0.25% of the initial PCE with encapsulation after 600 h at 85 °C and 85% humidity [115].

**Fig. 8:**
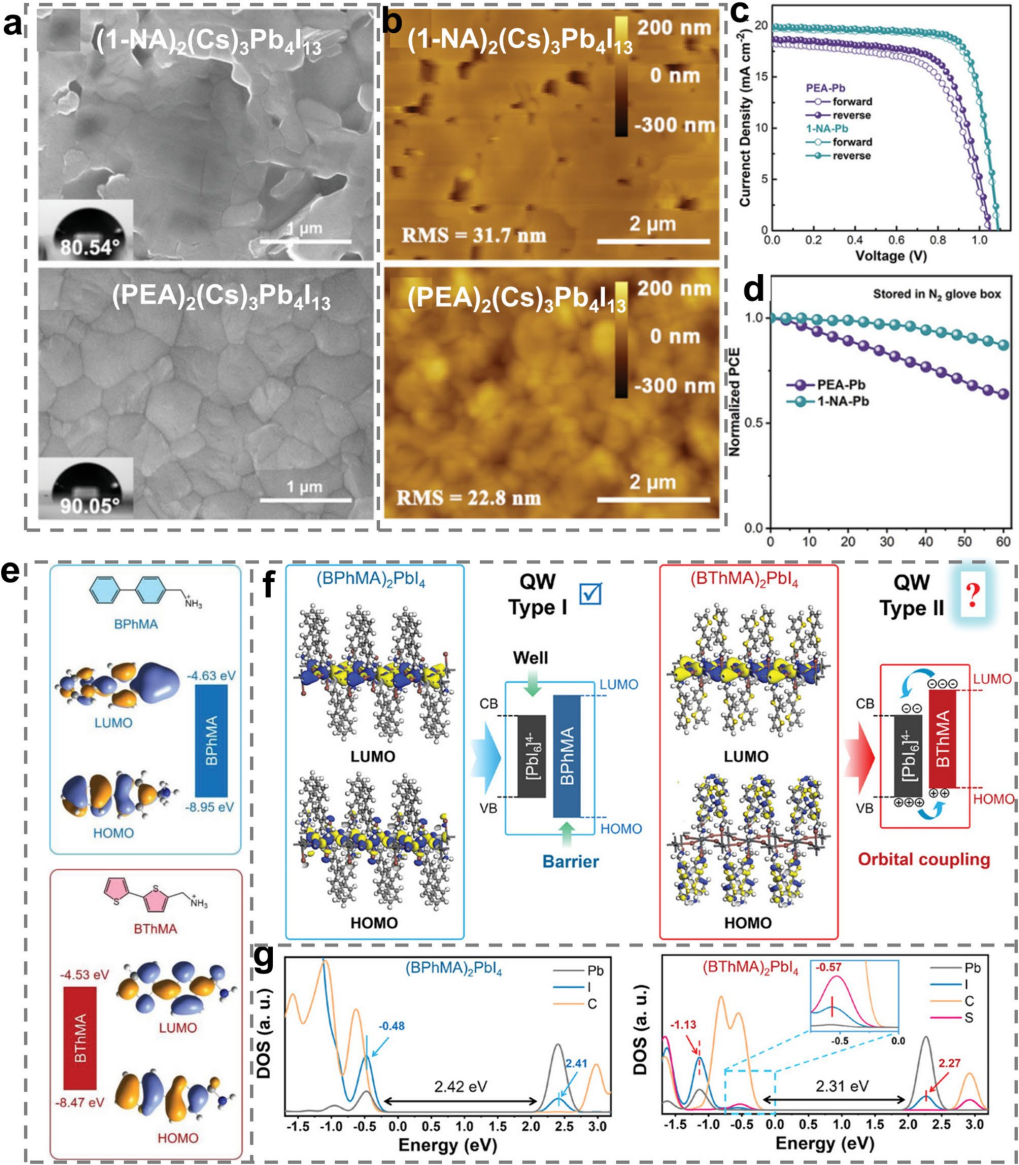
**a** Top-view SEM images and **b** AFM images of (1-NA)_2_(Cs)_3_Pb_4_I_13_ and (PEA)_2_(Cs)_3_Pb_4_I_13_ RPP films. **c** *J*-*V* curves and **d** stability of (1-NA)_2_(Cs)_3_Pb_4_I_13_ and (PEA)_2_(Cs)_3_Pb_4_I_13_ RP PSCs. Reproduced with permission [104]. Copyright 2022, Wiley–VCH GmbH. **e** Chemical structure and DFT-calculated energy levels of BPhMA and BThMA spacers. **f** DFT-calculated HOMO and LUMO of (BPhMA)_2_PbI_4_ and (BThMA)_2_PbI_4_ and proposed energy levels of organic and inorganic components. **g** Partial density of states plots of (BPhMA)_2_PbI_4_ and (BThMA)_2_PbI_4_. Reproduced with permission [176]. Copyright 2023, Wiley–VCH GmbH

The second way for extending the π-conjugated length is to construct the benzo structure, in which two or more aromatic rings connect each other by the shared C = C double bond or C–C single bond [122]. Many organic spacers such as 1-naphthylamine (1-NA) [104], naphthalenemethylammonium (NpMA) [174], 9-anthracenemethylammonium (AnMA) [174], benzimidazolium (Bn) [175], benzodithiophene (BDT) [122], and TTFA [83] have been developed for RPPs by using the benzo structure. Compared with PEA spacer, 1-NA spacer with longer π-conjugated length endowed RPPs with lower *E*_*b*_, more efficient separation of excitons, narrower *E*_*g*_ and faster interlayer charge transport. In addition, the benzo structure of 1-NA enhanced the inter-spacer interactions and HB interactions with inorganic frameworks, which contributed to improved film quality with lower defect density (Fig. 8a, b) [104]. Benefitting from these merits, (1-NA)_2_(Cs)_3_Pb_4_I_13_ RP PSCs yielded a high PCE of 16.62% with impressive stability (Fig. 8c, d) [104].

The third way to extend the π-conjugated length is similar to the second way and the difference lies in the linking way of two aromatic rings. To be specific, the third way is to build the biphenyl structure, in which the two aromatic rings connect each other via an independent -C–C- single bond. Based on this design principle, long-conjugated bithiopheneethylammonium (BThEA) [41], bithiophenemethylammonium (BThMA) [176], bibenzenemethylammonium (BPhMA) [176], phenylthiophenmethanamine (PTMA) [100] and CA [101] have been developed for 2D RPPs. Thanks to the appearance of S atoms, the BThMA spacer created more intensive orbital interactions with the inorganic octahedra, leading to the highest occupied molecular orbital (HOMO) of 2D RPPs to distribute at the conjugate skeleton of organic spacers. Thus, BThMA-based RPPs exhibited smaller *E*_*g*_ and more efficient charge transport (Fig. 8e-g). Additionally, the S atoms afforded BThMA-based RPP films with high crystallinity, large grain size and uniform morphology. As a result, (BThMA)_2_MA_4_Pb_5_I_16_ RP PSCs outperformed (BPhMA)_2_MA_4_Pb_5_I_16_ RP PSCs (the PCE of 18.05% vs 12.69%) [176]. The case of BThMA and BPhMA also evidences the importance of the strategy of introducing heteroatoms in the fused ring system.

Due to the different connection ways between two aromatic rings of the benzo and biphenyl structures, they present different structure characteristics. The biphenyl structure is more flexible than the benzo structure, while the more rigid benzo structure can give rise to stronger inter-spacer π···π interactions and spacer–inorganic framework interactions, which can affect the colloid chemistry of precursor and crystallization process. As we mentioned in the example of TTFA and BTFA spacer in Sect. 2.1, compared with BTFA with the biphenyl structure, the TTFA spacer better optimized the crystallization kinetics and contributed to high-quality RPP films, excellent optoelectronic properties and stability. Eventually, (TTFA)_2_MA_4_Pb_5_I_16_ RP PSCs achieved outstanding photovoltaic performance with a PCE of 19.41%, which is a champion for 2D PSCs with FA-based spacers [83].

Nonetheless, it is worth noting that the π-conjugated length should be extended appropriately and too long π-conjugated length may be counterproductive. This is because excessive π-conjugated length causes too large steric resistance, which will weaken HBs between spacer cations and inorganic frameworks and pose the risk of deteriorating the properties of RPPs for their photovoltaic applications. Xu et al.’s study confirms this idea through comparing NpMA and AnMA spacers. The NpMA spacer with two connected benzene rings can form stronger HBs with [PbI_6_]^4−^ octahedra framework than AnMA spacer. Under the influence of stronger HBs, NpMA-based RPPs grew along the direction perpendicular to the substrate, which extended carrier lifetime and promoted charge transport. By contrast, the AnMA spacer with three connected benzenes rings and longer π-conjugated length decreased the HB interaction with inorganic frameworks and brought about poor film quality. These characteristics further determined the photovoltaic performance of the corresponding RP PSCs. The PCE of NpMA-based RP PSCs (17.25%) was apparently higher than that of AnMA-based RP PSCs (14.47%) [174].

#### Strategy III: Enriching Functional Groups of the Ammonium Terminus

To directly tune the interaction between organic spacers and inorganic frameworks, an effective strategy is to enrich functional groups of the ammonium terminus since the ammonium terminus directly interact and connect inorganic frameworks. This strategy refers to that some other functional groups such as extra -NH- group, -F group and methyl group (-CH_3_) can be grafted onto the ammonium terminus, which can directly tune the interaction between organic spacers and inorganic octahedra, playing an important role in enhancing the stability of RPPs [73, 113, 130]. Besides, the introduction of other functional groups onto the ammonium terminus can affect the size, rigidity, the polarity and dipole moment of spacer cations, holding the potential to reduce the mismatch between organic layers and inorganic layers and promote charge transport [59, 177].

**Fig. 9:**
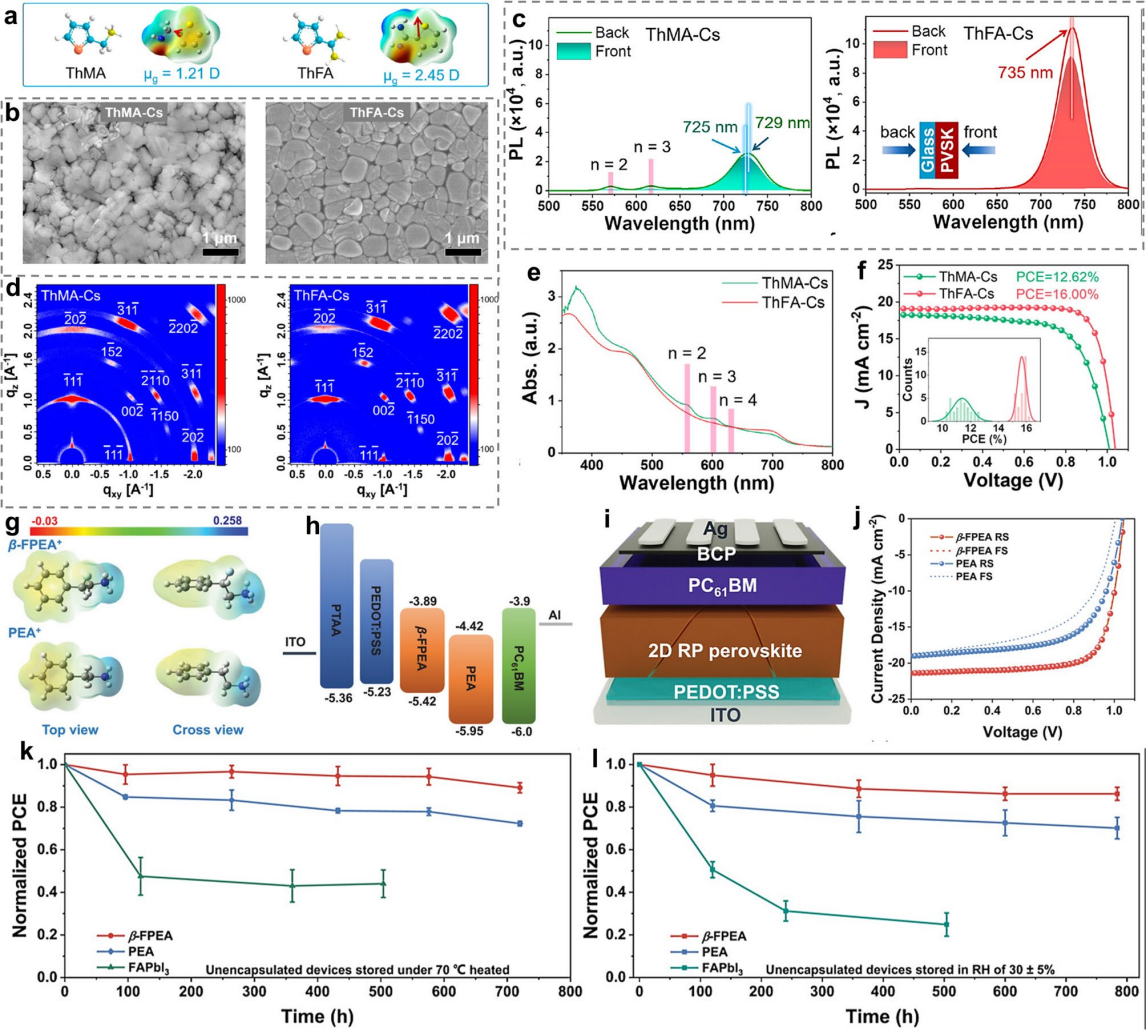
**a** Chemical structures of ThMA and ThFA spacers showing dipole moments. **b** SEM images of ThMA and ThFA-based RPP films. **c** PL spectra of ThMA and ThFA-based RPP films excited from front and back sides. **d** GIWAXS patterns and **e** UV–vis spectra of ThMA and ThFA-based RPP films. **f** *J*-*V* curves of ThMA and ThFA-based RP PSCs. Reproduced with permission [117]. Copyright 2022, American Chemical Society. **g** Top and cross views of the electrostatic potential maps of *β*-FPEA and PEA spacers. **h** Energy diagrams of *β*-FPEA- and PEA-based RP PSCs. **i** Device structure, **j** *J*-*V* curves and **k-l** stability test of *β*-FPEA- and PEA-based RP PSCs. Reproduced with permission [59]. Copyright 2023, Wiley–VCH GmbH

As for this strategy, the commonly utilized method is replacing the MA-based ammonium terminus with FA-based amidine terminus. In recent years, various FA-based organic spacers have been synthesized for novel RPPs [83, 117, 157, 178, 179]. Xu and co-workers investigated the influence of MA-based ammonium terminus and FA-based amidine terminus of spacers on CsPbI_3_-based RPPs. Compared with ThMA spacer, ThFA spacer (Fig. 9a) connected inorganic frameworks with multiple NH···I interactions, conferring CsPbI_3_ perovskites with obviously enhanced phase stability. In addition, the ThFA spacer also resulted in larger-size grains, more densely packed morphology (Fig. 9b), reduced small-n phases (Fig. 9c-e) and preferential vertical crystallization orientation (Fig. 9d), which suppressed charge recombination and promoted charge transport. As a consequence, ThFA-based RP PSCs (n = 5) delivered a PCE of 16.00%, while ThFA-based analogues only yielded a PCE of 12.62% (Fig. 9f) [117].

Besides, the -CH_2_- which links -NH_2_ group can be substituted to -NH-, forming a -NH-NH_2_ ammonium terminus. In the case of PHA, (PHA)_2_Cs_4_Pb_5_I_16_ RP PSCs achieved a performance of 16.23% with good environmental stability. The enhanced performance and stability for PHA-based RP PSCs compared with PA-based analogues could be mainly ascribed to the strengthened HBs between organic spacers and [PbI_6_]^4−^ octahedra, which increased the film crystallinity and stabilized the crystal structure [113]. Additionally, the -F group which are usually used to modify the non-ammonium terminus has been reported to decorate the ammonium terminus. The newly designed *β*-FPEA spacer exhibited larger dipole moment and stronger interactions with inorganic octahedra than PEA (Fig. 9g). Also, *β*-FPEA-based RPPs displayed more optimized energy level alignment (Fig. 9h) and better film quality. All these advantages conferred *β*-FPEA-based quasi-2D RP PSCs (n = 5) (Fig. 9i) with a PCE of 16.77% (Fig. 9j) and charming moisture and heat stability (Fig. 9k, l). The PCE was further improved to 19.11% by optimizing the hole transporting layer (HTL) materials [59].

Apart from the above-mentioned cases in which modifying the ammonium terminus of organic spacers intensified the spacer–inorganic framework interaction, some modification of ammonium terminus may weaken the spacer–inorganic framework interaction. For example, using -CH_3_ group to replace H of -NH_3_ group will deduct the HBs between organic spacers and inorganic frameworks. Compared with phenylammonium (PA, primary ammonium), N-phenylmethylammonium (PMA, secondary ammonium) and N,N-dimethylphenylammonium (DPA, tertiary ammonium), phenyltrimethylammonium (PTA, quaternary ammonium) with the minimum HB led to the best PR PSC performance, which has been attributed to the improved defect passivation ability by three -CH_3_ groups of PTA [130]. However, (PTA)_2_(MA)_3_Pb_4_I_13_ RP PSCs delivered a PCE of only 11.53% with the assistance of MACl additive, which is obviously inferior than that of the above-mentioned examples. We speculate that the relatively poor efficiency of PTA-based RP PSCs may be ascribed to the weak spacer–inorganic framework interaction, which increases *E*_*b*_ and is unfavorable for charge transport. Such discrepancies also demonstrate that modifying the ammonium terminus of spacers to strengthen the spacer–inorganic framework interaction is more recommended for realizing high-efficiency RP PSCs.

#### Strategy IV: Functionalizing the Non-Ammonium Terminus

To reduce the VdW gap between adjacent organic layers, it is of great importance to strengthen the inter-spacer interactions. The key of this goal lies in functionalizing the non-ammonium terminus via diverse functional groups, which can generate strong non-covalent interactions between neighboring organic layers in addition to the weak VdW interaction [77, 78]. These strong non-covalent interactions can not only enhance the stability of crystal structure but also benefit to charge transport and optimize the film formation process [78, 180]. Moreover, it is also possible for functional groups at the non-ammonium terminus to interact with the inorganic components, which can engage in modulating the film crystallization process and passivating defects [57, 79].

Under the guidance of this strategy, multifarious functional groups such as the halogen polar group, -COOH, -OH, and -S- group have been employed to modify the non-ammonium terminus of organic spacers for regulating RP PSC photovoltaic applications [156, 177, 179, 181–183]. Among them, the -F group seems to be the most popular one which have been applied for various types of organic spacers, probably owing to the strong hydrophobicity, large electronegativity and intense electron-withdrawing effect of -F group [70, 156, 184, 185]. Many spacers including 4-fluorophenethylammonium (F-PEA, 4FPEA or *p*F1PEA) [141, 156, 186, 187], 3-fluoro-benzyl ammonium (3FBA, *m*FBA) [182, 188], 4-fluorobenzylamine (*p*-FPMA, *p*FPBA, F-BZA, FBA) [180, 188, 189], 4-fluorobenzenaminium (FPA) [148], para-fluorobenzamidine (*p*-FPhFA) [190] and F3EA [70] have been developed by employing the -F group to modify the aromatic and alkyl non-ammonium terminus.

**Fig. 10:**
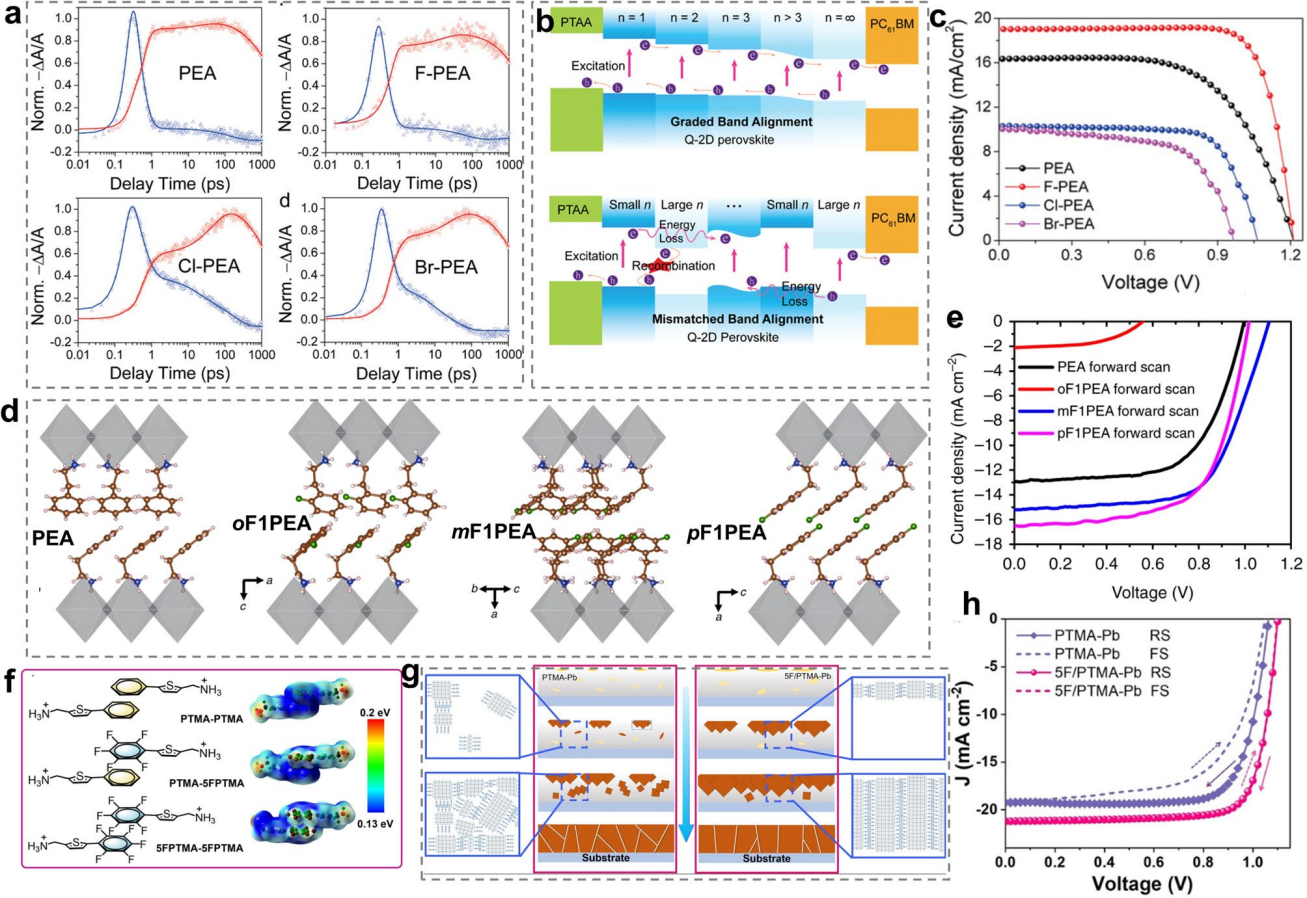
**a** TA kinetics probed at n = 4 (blue line) and n = ∞ (red line) bands under top excitation for PEA, F-PEA, Cl-PEA, and Br-PEA-based RPP films. **b** Schematic diagram of charge transport pathway based on graded band alignment and mismatched band alignment. **c** Influence of different halogen groups modified spacers on the photovoltaic performance of the resulting RP PSCs. Reproduced with permission [141]. Copyright 2020, Wiley–VCH GmbH. The influence of -F group substitution position on **d** intermolecular π-π packing motif and **e** photovoltaic performance of RP PSCs. Reproduced with permission [186]. Copyright 2019, Springer Nature. **f** Schematic illustrations and ESP maps of PTMA and 5FPTMA spacer arrays. **g** Schematic diagram showing the nucleation and crystallization mechanism of PTMA-Pb and 5F/PTMA-Pb RPP films. **h** *J*-*V* curves of PTMA-Pb and 5F/PTMA-Pb-based RP PSCs. Reproduced with permission [100]. Copyright 2024, Wiley–VCH GmbH

It seems that the -F group substitution exhibits greater advantage in optimizing the phase composition and distribution compared with other halogen groups (-Cl, -Br). Wang et al. demonstrated that the fluorinated PEA (F-PEA) spacer conferred RPPs with the orderly distribution of different n phases, resulting in the graded band alignment, which is beneficial for charge transport. By contrast, chlorinated and brominated PEA (Cl-PEA and Br-PEA) spacers brought about the random distribution, leading to the mismatched band alignment, which is detrimental to charge transport and separation (Fig. 10a-c). Accordingly, (F-PEA)_2_MA_3_Pb_4_I_12_ RP PSCs delivered a PCE of 18.10%, remarkably higher than that for PEA (12.23%), Cl-PEA (7.93%) and Br-PEA (6.08%)-based RP PSCs [141]. In spite of the low PCE detected for Cl-PEA-based RP PSCs, 4-chlorobenzylammonium (CBA) spacer overperformed FBA when using as a secondary spacer to optimize 2-hydroxyethylamine (HEA)-based quasi-2D RPPs in another study [183]. This observation implies that the function of organic spacers cannot be underestimated even though they do not perform well when serving as a unary spacer.

In addition, the presence of -F group in spacer cations is capable of strengthening the HB interaction between organic spacers and inorganic slabs and facilitating charge transport. For instance, compared with the unfluorinated benzamidine (PhFA) spacer, the para-fluorobenzamidine (*p*-FPhFA) spacer was founded to form stronger HBs with [PbI_6_]^4−^ octahedra. The resulting (*p*-FPhFA)_2_MA_n-1_Pb_n_I_3n+1-x_Cl_x_ RPP film exhibited better film quality with enlarged grain size and desirable crystal growth orientation, which achieved more efficient charge transport and longer carrier lifetime. In consequence, the PCE of (*p*-FPhFA)_2_MA_n-1_Pb_n_I_3n+1-x_Cl_x_ RP PSCs (17.37%) surpassed that of (FPhFA)_2_MA_n-1_Pb_n_I_3n+1-x_Cl_x_ PSCs (12.92%) [190].

Note that the position of functional groups on aromatic rings has a great influence on properties of organic spacers, which further affect the structure, film quality, optoelectronic properties of RPPs [186, 188]. In spite of only 6.08% of PCE for Br-PEA RP PSCs in the aforementioned case, a water-stable quasi-2D RP PSCs with the PCE of 18.20% was reported by using 3-bromobenzylammonium (3BBA) as spacer which enabled the formation of energetically ordered and highly crystalline RPP films. Such a difference may be related to the different substitution positions of -Br group on benzene ring as well as the smaller size of 3BBA spacers compared with Br-PEA. The influence of substitution position of the -F group on benzene ring has been explored in detail. According to the substitution position of -F group on the benzene ring, the monofluorinated PEA includes three types, 2-fluorophenethylammonium (*o*F1PEA), 3-fluorophenethylammonium (*m*F1PEA) and *p*F1PEA. The F substitution position can modulate the intermolecular π-π packing motif for *o*F1PEA, *m*F1PEA and *p*F1PEA, which tend to adopt edge-to-face stacking, herringbone configuration and parallel-displaced packing fashion, respectively (Fig. 10d). The different intermolecular π-π packing motif will result in different ordering of the organic bilayer. Comparatively, *o*F1PEA with the edge-to-face packing motif led to the most orientational disorder, which may further negatively influence the phase composition, film quality and photovoltaic performance. By contrast, *p*F1PEA with the parallel-displaced packing fashion showing the most orientational order is apt to produce better film morphology and higher photovoltaic performance (Fig. 10e) [186]. Not only for fluorinated PEA, *x*-fluorobenzylammonium (*p*FBA, *m*FBA, *o*FBA) is also coincident with such a rule that* p*FBA-based RP PSCs outperformed other counterparts [188]. As a matter of fact, the para substitution of -F group is the most commonly used tactic to design monofluorinated aromatic spacers, which may be ascribed to better film morphology and charge transport properties caused by the unique π-π packing fashion of para-fluorinated aromatic spacers [148, 156, 191, 192].

In addition to the substitution position of -F group on aromatic rings, the fluorination degree (the number of -F groups) also affects properties of organic spacers and the resulting RPPs [70]. Wang et al. indicated that increasing the fluorination degree of spacers can enhance the dipole moments of spacers and the formation energy of 2D RPPs. Even though the high dipole moment can promote the exciton separation, the large formation energy of 2D RPPs would result in the larger energy barriers to form good quality crystals and the tendency to form small domains [193]. Thus, it is necessary to balance the exciton and charge properties as well as film quality by appropriately controlling the fluorination degree in order to maximize photovoltaic performance of RP PSCs. By a comprehensive consideration, difluorine substitution may be the most desirable. Recently, some difluorinated spacers such as DF-BZA [58], 3,3-DFAz [128] and DFP [31] have been developed for high-performance RP PSCs. Both of them exhibited high dipole moments and endowed superior RPP film quality and brilliant optoelectronic properties. Ultimately, (DF-BZA)_2_FA_3_Pb_4_I_13_, (3,3-DFAz)_2_FA_3_Pb_4_I_13_ and (DFP)_2_MA_4_Pb_5_I_16_ RP PSCs achieved a brilliant photovoltaic performance with PCE of 19.24%, 19.26%, and 19.43%, respectively [31, 58, 128].

From the perspective of generating stronger π-π interactions between aromatic spacers, the fluorination degree can be further improved to perfluorination of benzene rings. However, the perfluorinated spacer needs to be utilized with the unfluorinated analogue in combination. Due to the different electronegativities of perfluorinated and unfluorinated benzene rings, the strong quadrupole–quadrupole interaction, which is also termed as the aryl-perfluoroaryl interaction, can form between them, playing a critical role in regulating molecular packing and film quality [152, 194]. Very recently, perfluoro-phenylthiophenmethanamine iodide (5FPTMAI) spacer has been successfully developed and used in combination with PTMA to fabricate high-quality 2D RPP films (Fig. 10f). With assistance of strong quadrupole–quadrupole interaction between PTMA and 5FPTMAI, 5F/PTMA-Pb films realized a top-down film growth process (Fig. 10g), effectively suppressing nucleation in the bulk phase and decreasing intra-aggregate grain boundaries, thereby reducing defects and inhibiting charge recombination. As a result, ((5FPTMA)_0.1_(PTMA)_0.9_)_2_MA_4_Pb_5_I_16_ RP PSCs delivered a PCE of 18.56% (Fig. 10h) [100].

Apart from the -F group, the -COOH group and -S- group have also gained great attention to modify the non-ammonium terminus of organic spacers [57, 77–79, 195]. For example, the aforementioned GABA, GPA, Gly and MTEA spacers have been developed according to this strategy. The presence of -COOH and -S- group at the non-ammonium terminus resulted in the formation of HB and S–S interactions between adjacent organic layers, which facilitated charge transport dynamics and optimized the crystallization process. Therefore, all these spacers contributed to outstanding performance of RP PSCs [57, 77–79, 195]. More intriguingly, -COOH group-modified spacers and their derivatives such as acylamino group (-CO-NH_2_) and ester group (-COO-)-modified spacers are conductive to realize the self-additive effect, which can not only manipulate the crystallization kinetics but also manage bulk defects. These effects can jointly give rise to highly-efficient and stable RP PSCs [57, 79]. In terms of the self-additive capability, a series of glycine (Gly)-based spacer cations, including Gly, glycinamide (Gly-A), and glycine ethyl ester (Gly-E) have been studied and compared. Gly-E showed the best self-additive effect and endowed RP PSCs with the highest PCE of 21.60% and impressive stability [57].

#### Strategy V: Adjusting the Chain Length and Size of Organic Spacers

In consideration of the multiple influences of organic spacer size, there is a need to reasonably and precisely adjust the chain length and size of organic spacers to balance the tunneling effect, steric effect, hydrophobicity and film quality. In this section, we present three methods to directly tune the chain length and size of organic spacers.

**Fig. 11:**
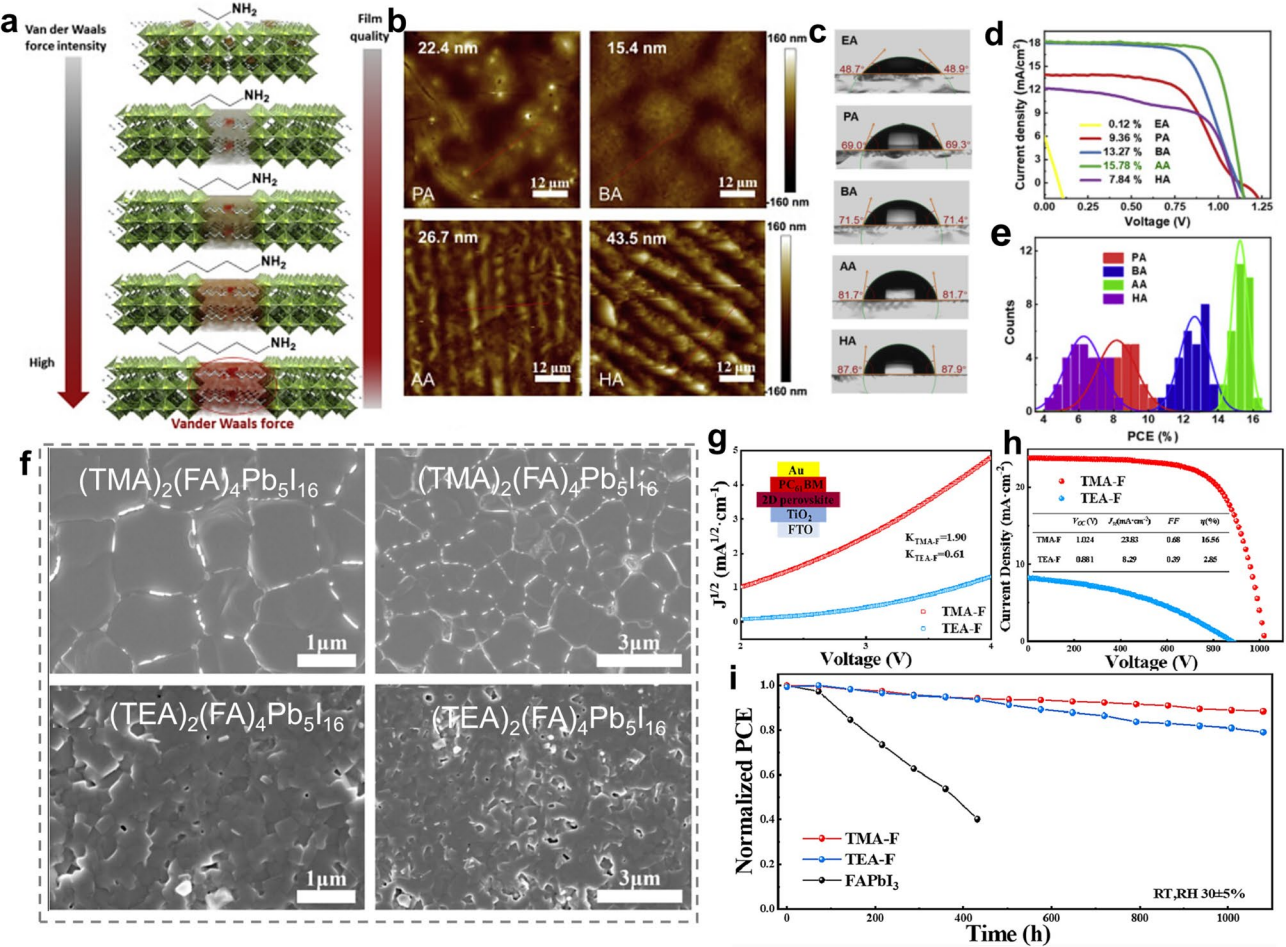
**A** Schematic illustration of crystal structure of RPPs using spacers with different chain length from EA to HA, showing the influence of different chain length on VdW force. **b** AFM height images and **c** water surface CAs for RPP films using spacers with different chain length from EA to HA. **d** *J*-*V* curves and **e** PCE distribution results for the corresponding RP PSCs. Reproduced with permission [71]. Copyright 2021, Elsevier Inc. **f** Top-view SEM images and **g** electron mobility for (TEA)_2_(FA)_n-1_Pb_n_I_3n+1_ and (TEA)_2_(FA)_n-1_Pb_n_I_3n+1_ films. **h** *J*-*V* curves and **i** long-term stability of (TEA)_2_(FA)_n-1_Pb_n_I_3n+1_ and (TEA)_2_(FA)_n-1_Pb_n_I_3n+1_ PSCs. Reproduced with permission [121]. Copyright 2024, American Chemical Society

The easiest way for adjusting the size of organic spacers is to control the number of straight-chain carbon, which is applicable for both alkylammonium and aromatic ammonium spacers [69, 71, 121, 196–198]. In addition to BA with four-carbon linear chain, n-propylamine (PA) with three-carbon linear chain has been reported as organic spacers for RP PSCs, which delivered a PCE of 10.41% via optimizing the precursor-solvent interaction [199]. Comparatively speaking, for straight alkylammonium spacers, increasing the chain length by increasing the carbon number can enhance the interspace VdW interactions (Fig. 11a), which could promote aggregations in perovskite precursor and boost the growth of 2D and 3D-like phase components toward the bottom and top area of perovskite films, respectively, eventually giving rise to preferably vertical phase distribution. By adjusting the carbon number from two to six, amylamine [AA] with five carbon atoms induced an intermediate aggregation and the optimal crystallization process, eventually affording RPPs the best photovoltaic performance (Fig. 11b-e) [71]. For aromatic ammonium spacers, it appears that the length of the alkylammonium chain on aromatic rings greatly affects the phase composition of RPPs and the performance of RP PSCs [121, 196]. Comparing TMA with TEA, it is surprisingly to find that the TMA spacer with shorter chain length inhibited the formation of low-n phases and gave rise to brilliant film quality and notably enhanced electron mobility, while the TEA spacer with longer chain length brought about more low-n phases, hindering charge transfer and decreasing light absorption (Fig. 11f, j). As a result, the PCE of (TMA)_2_(FA)_4_Pb_5_I_16_ RP PSCs reached 16.56%, almost seven times higher than that of (TEA)_2_(FA)_4_Pb_5_I_16_ RP PSCs [121].

In terms of the alkylammonium spacers with fixed carbon atoms number, two strategies have been utilized to adjust the size of these organic spacers. The first way is to change C–C single bond to C = C double bond or C≡C triple bond, which can minorly tune the chain length of alkylammonium spacers. Taking BA, 1-amino-3-butene (BEA) and butyn-1-amine (BYA) spacers as examples, the introduction of C = C bond resulted in the shortest chain length of BEY, which could increase the probability of charge tunneling and foster the charge transportation, thereby enhancing the photovoltaic performance [97]. The second route is to tune the steric configuration from the straight-chain shaped alkylammonium to branched-chain shaped alkylammonium, which is equivalent to reduce the length of the main straight chain. iso-BA spacer is a typical example in this respect, which has been reported to enhance the film crystallinity and light absorption and contribute to better RP PSC performance than BA spacer [200].

## Conclusions and Perspective

To best of our knowledge, 2D RPPs represent promising candidates for photovoltaic applications to tackle the ticklish problem of instability that is inevitable for 3D PSCs. However, RP PSCs suffer from inferior efficiency because of the large *E*_*b*_, the blocked charge transport and poor film quality of RPPs, all of which are associated with the presence of bulky organic spacer. Thus, it is essential to manage organic spacer to overcome these challenges for developing high-performance RP PSCs. By rationally designing the molecular structure of organic spacers, the crystallization kinetics, charge separation and transporting abilities as well as the crystal stability of RPPs can be well manipulated, holding great potential to achieve decent photovoltaic performance. The main focus of the design effort on organic spacers should be the steric configuration and dipole moments of organic spacers as well as the intermolecular interactions between organic spacers as these three aspects are closely related to the above-mentioned properties of RPPs. By a comprehensive consideration of these factors, five molecular structure design strategies are advised based on recent literature on spacer cation design engineering.

Strategy I is to introduce heteroatoms into fused ring system to optimize the polarity and dipole moment, thus achieving favorable interactions between the inter-spacer and spacer–inorganic frameworks. Strategy II is to appropriately extend the π-conjugated length, the main target of which is the aromatic spacer cations. This strategy can be implemented by three specific ways, namely introducing the C = C double bond, increasing the number of aromatic rings and adjusting their connection modes. This strategy can lead to a direct result of increasing π-π interactions between spacer cations, which further affect the charge transporting ability and film formation process.

Strategies III and IV are to functionalize spacer cations with special functional groups on different locations to suit various purposes. To be more specific, strategy III is to enrich the ammonium terminus via introducing functional groups and mainly aims to modulate the spacer–inorganic framework interaction. By contrast, the main purpose of Strategy IV is to reinforce the inter-spacer interaction and reduce the VdW gap by functionalizing the non-ammonium terminus. Noteworthy that the precise category of functional groups used to modify spacer cations should be carefully selected as some functional groups such as the methoxyl (-O-CH_3_) group may cause negative effects. Both 4-methoxyphenethylammonium (MeO-PEA) spacer and 4-methoxybenzylamine (p-MeOPMA) did not bring better RP PSC performance than PEA and PMA spacer [156, 189]. Additionally, the position of functional groups and the functionalization degree should be rationally controlled since these aspects would also affect the role-play of organic spacers.

Strategy V is about the precise management of chain length and size of organic spacer, which is the base of other strategies as the size of spacer cations should be taken into consideration no matter which strategy used to tailor the molecular structure of organic spacers. We put forward three pathways here to manage the chain length and size of spacer cations, including adjusting the number of carbon atoms, introducing C = C double bond or C≡C triple bond and transforming the straight-chain structure to branched-chain structure. The organic spacers designed by using these proposed strategies as well as the photovoltaic performance of corresponding RP PSCs are summarized in Table 1.

**Table 1:** Molecular structure design strategies of organic spacers and photovoltaic performance of the corresponding RP PSCs

Strategy	Spacer cation	RPP	Device architecture	PCE (%)	Stability	References
Strategy I: Introducing heteroatoms in the fused ring system	4-AEP	(4-AEP)_2_MA_4_Pb_5_I_16_	FTO/C60/RPP/Spiro-OmeTAD/Au	11.68	95% of PCE after experiencing 1000 h in ambient air (RT, 30% RH); without encapsulation	[81]
	TEA	(TEA)_2_MA_3_Pb_4_I_13_	ITO/PEDOT:PSS/RPP/PCBM/Bphen/Al	11.32	80% of PCE after 270 h storage in air with 60 ± 5% relative humidity at 25 °C; without encapsulation	[149]
	THFMA	(THFMA)_2_MA_3_Pb_4_I_13-x_Cl_x_	ITO/PEDOT:PSS/RPP/PCBM/BCP/Ag	13.79	80% of PCE after 934 h in N_2_; without encapsulation	[164]
	FuMA	(FuMA)_2_MA_3_Pb_4_I_13-x_Cl_x_		18	94% of PCE after 1850 h in N_2_; 83% of PCE after 768 h in N_2_ at 85 °C; 80% of PCE after 768 h under continuous light illumination (100 mW cm^−2^) in N_2_; without encapsulation	
	FuMA	(FuMA)_2_(MA)_4_Pb_5_I_16_	ITO/SnO_2_/RPP/Spiro-OmeTAD/Au	15.66	83.5% of PCE after 1800 h in ambient air (RT, 5%–10% RH); 94% of PCE after 1640 min at ambient atmosphere at 85 °C; without encapsulation	[161]
	ThMA	(ThMA)_2_(FA)_4_Pb_5_I_16_	ITO/PEDOT:PSS/RPP/PCBM/BCP/Ag	19.06	99% of PCE after 552 h in ambient conditions (RH, 30 ± 5%); ~ 96% of PCE after 576 h in N_2_ at 80 °C; without encapsulation	[201]
	ThFA	(ThFA)_2_(MA)_2_Pb_3_I_10_	ITO/PEDOT:PSS/RPP/PCBM/BCP/Ag	16.72	99% of PCE after 3000 h in N_2_; without encapsulation	[157]
	SeMA	(SeMA)_2_(FA)_4_Pb_5_I_16-x_Cl_x_	ITO/PEDOT:PSS/RPP/PDTL/PCBM/BCP/Ag	18.65	100% of PCE after 1008 h in ambient conditions (30 ± 5% RH); 86% of PCE in N_2_ at 60 °C; 97% of PCE after 1008 h continuous light soaking (100 mW cm^−2^) in N_2_; without encapsulation	[142]
	TPM	(TPM)_2_(MA)_3_Pb_4_I_13_	FTO/TiO_2_/RPP/Spiro-OmeTAD/Au	9.46	40% of PCE after 250 h in ambient conditions (RT, 35% ~ 50% RH); without encapsulation	[165]
	TPM, TFF	(TPM_0.75_TFF_0.2_)_2_(MA)_3_Pb_4_I_13_		10.01	80% of PCE after 250 h in ambient conditions (RT, 35% ~ 50% RH); without encapsulation	
	BM	(BM)_2−x_(BA)_x_MA_3_Pb_4_I_13_	ITO/SnO_2_/RPP/Spiro-OmeTAD/Au	17.3	87% of PCE after 100 h under RH 85% and 85 °C heating; 90% after 1000 h aging in air with RH 30% − 60%; 86% of PCE after 200 h under continuous 1 sun illumination in N_2_; without encapsulation	[172]
Strategy II: Appropriately extending the π-conjugated length	PPA	(PPA)_2_(Cs_0.05_(FA_0.88_MA_0.12_)_0.95_)_3_Pb_4_(I_0.88_Br_0.12_)_13_	ITO/PTAA/RPP/C60/BCP/Cu	14.76	93.8 ± 0.25% of PCE with encapsulation after 600 h at 85 °C and 85% humidity; without encapsulation	[115]
	1-NA	(1-NA)_2_(Cs)_3_Pb_4_I_13_	FTO/TiO_2_/RPP/Spiro-OmeTAD/MoO_3_/Ag	16.62	87.1% of the initial PCE after 60 days of storage in N_2_; without encapsulation	[104]
	BThFA	(BTFA)_2_MA_4_Pb_5_I_16_	ITO/PEDOT:PSS/RPP/PCBM/Ag	15.43	78% of PCE after 588 h under continuous light illumination (95 ± 5 mW cm^−2^) in N_2_; without encapsulation	[83]
	TTFA	(TTFA)_2_MA_4_Pb_5_I_16_		19.41	90% of PCE after 777 h under continuous light illumination (95 ± 5 mW cm^−2^) in N_2_; without encapsulation	
	NpMA	(NpMA)_2_(MA)_3_Pb_4_I_13_	ITO/PEDOT:PSS/RPP/PCBM/BCP/Ag	17.25	91% of PCE after 4100 h in N_2_ at RT; 89% of PCE after 1000 h in ambient conditions with 30 ± 5% RH; 80% of PCE after 600 h in N_2_ at 80 °C; 79% of PCE after 600 h in N_2_ under continuous light illumination; without encapsulation	[174]
	AnMA	(AnMA)_2_(MA)_3_Pb_4_I_13_		14.47	90% of PCE after 4100 h in N_2_ at RT; 80% of PCE after 1000 h in ambient conditions with 30 ± 5% RH; 77% of PCE after 600 h in N_2_ at 80 °C; 76% of PCE after 600 h in N_2_ under continuous light illumination; without encapsulation	
	Bn	(Bn)_2_(MA)_9_Pb_10_I_31_	ITO/s-SnO_2_/c-SnO_2_/RPP/Spiro-OmeTAD/MoO_3_/Ag	15.12	92.1% of PCE after 720 h in air (40% − 45% relative humidity); 87.9% of PCE after 528 h in N_2_ at 85 C; without encapsulation	[175]
	BDT	(BDT)_2_MA_99_Pb_100_N_301_	FTO/TiO_2_/RPP/Spiro/Au	14.67	64% of PCE after 130 h under constant one-sun illumination in N_2_	[122]
	BPhMA	(BPhMA)_2_MA_4_Pb_5_I_16_	ITO/PEDOT:PSS/RPP/PCBM/BCP/Ag	12.69	84.9% of PCE after 670 h under ambient conditions (RH = 45 ± 5%)	[176]
	BThMA	(BThMA)_2_MA_4_Pb_5_I_16_		18.05	88.6% of PCE after 670 h under ambient conditions (RH = 45 ± 5%)	
	BThEA	(BThEA)_2_(MA)_6_Pb_7_I_22_	ITO/PEDOT:PSS/RPP/PCBM/BCP/Ag	13.3	NA	[41]
	PTMA	(PTMA)_2_MA_4_Pb_5_I_16_	ITO/PEDOT:PSS/RPP/PCBM/BCP/Ag	15.66	82% of PCE after 936 h in N_2_ at 70 °C; 75% of PCE after 873 h under continuous light soaking (100 mW cm^2^); without encapsulation	[100]
	CA	CA_2_MA_4_Pb_5_I_16_	ITO/SnO_2_/RPP /Spiro-OMeTAD/Au	18.23	85% after 2000 h in in an atmospheric environment with 65% RH and 25 °C; 95% after 312 h under 1-sun illumination; 95% after 168 h 70 °C heat treatment; without encapsulation	[101]
Strategy III: Enriching functional groups of the ammonium terminus	ThFA	(ThFA)_2_Cs_4_Pb_5_I_16_	ITO/PEDOT:PSS/RPP/PCBM/BCP/Ag	16	98% of PCE after 3000 h in N_2_; 92% of PCE after 960 h in N_2_ at 80 °C; 94% of PCE after 960 h under continuous light soaking (100 mW cm^−2^) in N_2_; without encapsulation	[117]
	ThMA	(ThMA)_2_Cs_4_Pb_5_I_16_		12.62	96% of PCE after 3000 h in N_2_; 88% of PCE after 960 h in N_2_ at 80 °C; 91% of PCE after 960 h under continuous light soaking (100 mW cm^−2^) in N_2_; without encapsulation	
	CPFA	(CPFA)_2_MA_8_Pb_9_(I_0.857_Cl_0.143_)_28_	FTO/TiO_2_/RPP/Spiro-OmeTAD/Au	14.78	~ 80% of PCE after over 2000 h under ambient conditions (RT, RH: 35%); without encapsulation	[179]
	GA	(F-PEA_0.8_GA_0.2_)_2_MA_3_Pb_4_I_13_	ITO/PTAA/RPP/C60/BCP/Cu	17.50	87% of PCE after 500 h in the ambient condition (40%–50% relative humidity); 90% of PCE after 1000 h under continuous one-sun equivalent illumination in N_2_	[73]
	PHA	(PHA)_2_Cs_4_Pb_5_I_16_	FTO/TiO_2_/RPP/Spiro-OmeTAD/Ag	16.23	~ 70% of PCE after 30 h in N_2_ at 80 °C; 50% of PCE after 40 h in the humidity of 30–35%; without encapsulation	[113]
	*β*-FPEA	(β-FPEA)_2_(FA)_4_Pb_5_I_16_	ITO/PTAA/PEDOT:PSS/RPP/PCBM/Al	16.77	~ 90% of PCE after 720 h in N_2_ at 70 °C; 80% of PCE after 780 h of storage in ambient conditions (RH, 35 ± 5%); without encapsulation	[59]
	PA	(PA)_2_(MA)_3_Pb_4_I_13_	ITO/SnO_2_/RPP/Spiro-OmeTAD/Ag	3.9	NA	[130]
	PMA	(PMA)_2_(MA)_3_Pb_4_I_13_		7.8	NA	
	DPA	(DPA)_2_(MA)_3_Pb_4_I_13_		4.3	NA	
	PTA	(PTA)_2_(MA)_3_Pb_4_I_13_		11.53	90% of PCE after 1600 h in N_2_	
Strategy IV: Functionalizing the non-ammonium terminus	F-PEA	(F-PEA)_2_(MA)_4_Pb_5_I_16_	ITO/PEDOT:PSS/RPP/PC61BM/BCP/Ag	14.5	90% of PCEs after 40 d in air with a humidity of 40%–50%; 97% of PCE after 3 h in N_2_ under AM 1.5G illumination; without encapsulation	[156]
	MeO-PEA	(MeO-PEA)_2_(MA)_4_Pb_5_I_16_		9.9	97% of PCEs after 40 d in air with a humidity of 40%–50%; 97% of PCE after 3 h in N_2_ under AM 1.5G illumination; without encapsulation	
	3BBA	(3BBA)_2_MA_n-1_Pb_n_I_3n+1-x_Cl_x_(3 < n < 4)	ITO/PTAA/RPP/PCBM/Cr/Ag	18.2	82% of PCE after 2400 h in a chamber at room temperature with a relative humidity of ≈40%; without encapsulation	[177]
	FPA	(FPA)_2_Cs_4_Pb_5_I_16_	FTO/TiO_2_/RPP/Spiro-OmeTAD/MoO_3_/Ag	15.18	70% of PCE after 100 h in humid condition with RH ~ 25%–30%; 70% of PCE after 96 h in N_2_ condition at 80 °C; without encapsulation	[148]
	F-PEA	(F-PEA)_2_MA_3_Pb_4_I_13_	ITO/PTAA/RPP/PCBM/PEI/Ag	18.1	over 90% after 30 days in humidity air at 40%-50% RH; 80% of PCE after 30 days in N_2_ at 80 °C; without encapsulation	[141]
	Cl-PEA	(Cl-PEA)_2_MA_3_Pb_4_I_13_		7.93	over 90% after 30 days in humidity air at 40%-50% RH; 30% of PCE after 15 days in N_2_ at 80 °C; without encapsulation	
	Br-PEA	(Br-PEA)_2_MA_3_Pb_4_I_13_		6.08	over 90% after 30 days in humidity air at 40%-50% RH; 20% of PCE after 12 days in N_2_ at 80 °C; without encapsulation	
	4FPEA	(4FPEA)_2_(MA)_4_Pb_5_I_16_	ITO/PTAA/RPP/PCBM/PEI/Ag	17.34	nearly 93% of PCE after 500 h under ambient air with a relative humidity of 55%–65%; almost 100% of PCE after 500 h in N_2_ at 55 °C; without encapsulation	[184]
	4FPEA	(4FPEA)_2_(FA)_4_Pb_5_I_16_	ITO/PTAA/RPP/PCBM/BCP/Ag	21.07	90% of PCE after 3000 h in 80 ± 5% RH moist air environment (25–30 °C) with encapsulation; 90% of PCE after 1000 h in the humid environment with 40 ± 5% RH; without encapsulation	[16]
	F-PEA	(F-PEA)_2_MA_4_Pb_5_I_16_	FTO/TiO_2_/RPP/Spiro-OmeTAD/Au	13.64	65% of PCE after 576 h in ambient environment at 70 °C; without encapsulation	[166]
	HEA	(HEA)_2_(Cs_0.1_FA_0.9_)_8_Pb_9_(I_0.95_Br_0.05_)_28_	FTO/c-TiO_2_/m-TiO_2_/RPP/Spiro-OmeTAD/Au	15.19	55% of PCE after aging at 45 ± 5% RH in dark for 1500 h; without encapsulation	[183]
	CBA	(HEA_0.9_CBA_0.1_)_2_(Cs_0.1_FA_0.9_)_8_Pb_9_(I_0.95_Br_0.05_)_28_		17.67	90% of PCE after aging at 45 ± 5% RH in dark for 1500 h; without encapsulation	
	FBA	(HEA_0.9_FBA_0.1_)_2_(Cs_0.1_FA_0.9_)_8_Pb_9_(I_0.95_Br_0.05_)_28_		18.75	85% of PCE after aging at 45 ± 5% RH in dark for 1500 h; without encapsulation	
	*o*F1PEA	(*o*F1PEA)_2_MA_3_Pb_4_I_13_	ITO/PEDOT:PSS/RPP/PCBM/BCP/Al	0.73	N/A	[186]
	*m*F1PEA	(*m*F1PEA)_2_MA_3_Pb_4_I_13_		10.17		
	*p*F1PEA	(*p*F1PEA)_2_MA_3_Pb_4_I_13_		10.89		
	*o*FBA	(*o*FBA)_2_MA_4_Pb_5_I_16_	ITO/PTAA/RPP/PCBM/BCP/Ag	12.89	60.64% of PCE after 600 h under the ambient environment with 60 ± 5% humidity at 25 °C; without encapsulation	[188]
	*m*FBA	(*m*FBA)_2_MA_4_Pb_5_I_16_		14.67	71.91% of PCE after 600 h under the ambient environment with 60 ± 5% humidity at 25 °C; without encapsulation	
	*p*FBA	(*p*FBA)_2_MA_4_Pb_5_I_16_		17.12	83.13% of PCE after 600 h under the ambient environment with 60 ± 5% humidity at 25 °C; without encapsulation	
	DF-BZA	(DF-BZA)_2_FA_3_Pb_4_I_13_	ITO/PTAA/RPP/PCBM/BCP/Ag	19.24	over 90% after ≈1400 h in N_2_ at 85 °C; 90% of PCE after 1100 h in ambient air with 30 ± 5% RH; without encapsulation	[56]
	3-OHAz	(3-OHAz)_2_MA_3_Pb_4_I_13-x_Cl_x_	ITO/PEDOT:PSS/RPP/PCBM/BCP/Ag	11.35	70% of PCE after 1104 h in N_2_ at 60 °C; 80% of PCE after 750 h in ambient air with a RH of 35 ± 5%; without encapsulation	[58]
	3,3-DFAz	(3,3-DFAz)_2_MA_3_Pb_4_I_13-x_Cl_x_		19.28	83% of PCE after 1104 h in N_2_ at 60 °C; 90% of PCE after 888 h in ambient air with a RH of 35 ± 5%; without encapsulation	
	DFP	(DFP)_2_MA_4_Pb_5_I_16_	FTO/SnO_2_/RPP/Spiro-OmeTAD/Ag	19.43	86% of PCE after 2300 h under natural conditions; 93% of PCE after 520 h in N_2_ at 65 °C; 95% of PCE after 500 h of continuous illumination in N_2_ at 65 °C; without encapsulation	[31]
	F3EA	((BA)_0.94_(F3EA)_0.06_)_2_(MA)_3_Pb_4_I_13_	ITO/PEDOT:PSS/RPP/PCBM/BCP/Ag	12.51	80% of PCE after 260 h in N_2_; without encapsulation	[70]
	5FPTMA	(5FPTMA_0.1_PTMA_0.9_)_2_MA_4_Pb_5_I_16_	ITO/PEDOT:PSS/RPP/PCBM/BCP/Ag	18.56	92% of PCE after 936 h in N_2_ at 70 °C; 93% of PCE after 873 h under continuous light soaking (100 mW cm^−2^); without encapsulation	[100]
	p-FPhFA	(p-FPhFA)_2_MA_4_Pb_5_I_16−x_Cl_x_	ITO/PEDOT:PSS/RPP/PCBM/BCP/Ag	17.37	99% of PCE after 3000 h in N_2_; 82% of PCE after 450 h in N_2_ under continuous light soaking (100 mW cm^−2^); without encapsulation	[190]
	phFA	(PhFA)_2_MA_4_Pb_5_I_16−x_Cl_x_		12.92	68% of PCE after 3000 h in N_2_; 77% of PCE after 450 h in N_2_ under continuous light soaking (100 mW cm^−2^); without encapsulation	
	GABA	(GABA)_2_MA_3_Pb_4_I_13_	ITO/SnO_2_/RPP/Spiro-OmeTAD/Au	18.73	over 93% of its initial PCE after 1200 h under ambient conditions (50 ± 10% relative humidity, RT); 95% of PCE after 500 h after 500 h under 85% relative humidity in the humidity chamber (RT); 92.8% of PCE after 1000 h under illumination in the N_2_; without encapsulation	[78]
	GPA	(GPA)_2_(MA)_4_Pb_5_I_16_	ITO/PEDOT:PSS/RRP/PCBM/BCP/Ag	17.71	~ 96% of PCE after 1100 h of continuous light operation in a N_2_ glove box with a humidity of ≈10%; 93% of PCE after 360 h under continuous heating at 65 °C; 90% of PCE after 4800 h in N_2_; without encapsulation	[77]
	GPA	(GPA_0.85_FPEA_0.15_)_2_MA_4_Pb_5_I_16_	ITO/PEDOT: PSS/RRP/PCBM/BCP/Ag	18.37	87% of PCE after 2800 h of aging under N_2_-filled conditions (relative humidity 25 ± 10% at 25 °C); 93.2% of PCE after 400 h in N_2_ at 70 °C	[195]
	Gly	Gly_2_(Cs_0.05_FA_0.95_)_3_Pb_4_I_13_Cl_2_]_0.9_(FAPbBr_3_)_0.1_	FTO/bl-TiO_2_/mp-TiO_2_/RPP/Spiro-OmeTAD/Au	15.61	94% of PCE after 2000 h under about 50% RH; after 240 h of thermal aging test at 85 °C; 66% of PCE after 20 h under UV light for 20 h	[79]
	Gly	(Gly)_2_FA_5_Pb_6_I_17_Cl_2_	ITO/SnO_2_/RPP/Spiro-OmeTAD/Au	16.79	83% of PCE after 4320 h in a humidity environment (45 ± 5% RH); 82% PCE after 480 h at 85 °C and 10% RH; 65% of PCE after 760 h under continuous light exposure; without encapsulation	[57]
	Gly-A	(Gly-A)_2_FA_5_Pb_6_I_17_Cl_2_		18.66	85% of PCE after 4320 h in a humidity environment (45 ± 5% RH); 83% PCE after 480 h at 85 °C and 10% RH; 68% of PCE after 760 h under continuous light exposure; without encapsulation	
	Gly-E	(Gly-E)_2_FA_5_Pb_6_I_17_Cl_2_		21.6	93% of PCE after 4320 h in a humidity environment (45 ± 5% RH); 89% PCE after 480 h at 85 °C and 10% RH; 77% of PCE after 760 h under continuous light exposure; without encapsulation	
Strategy V: Adjusting the chain length and size of organic spacers	PA	(PA)_2_MA_3_Pb_4_I_13_	ITO/PTAA/RPP/C60/BCP/Ag	9.36	N/A	[71]
	BA	(BA)_2_MA_3_Pb_4_I_13_		13.27	N/A	
	AA	(AA)_2_MA_3_Pb_4_I_13_		15.78	99.34% of PCE after 1848 h in N_2_; 99.9% of PCE after 744 h in ambient air conditions (65% humidity); without encapsulation	
	HA	(HA)_2_MA_3_Pb_4_I_13_		7.84	N/A	
	PA	(PA)_2_MA_4_Pb_5_I_16_	FTO/TiO_2_/RPP/Spiro-OmeTAD/Au	10.41	98% of PCE after 500 h in air (in the dark, relative humidity ∼50 − 60% RH, RT); without encapsulation	[199]
	OA	(BA_0.97_OA_0.03_)_2_MA_3_Pb_4_I_13_	ITO/PEDOT:PSS/ RPP/PCBM/BCP/Ag	11.90	63% of PCE after 410 h in air with a humidity of 30 ± 5% and a temperature of 20 ± 5 °C; 93% of PCE after 410 h in N_2_	[198]
	TMA	(TMA)_2_(FA)_4_Pb_5_I_16_	FTO/TiO_2_/RPP/Spiro-OmeTAD/Au	16.56	88.4% of PCE after 1080 h under environmental conditions (RT, RH = 30 ± 5%); without encapsulation	[121]
	TEA	(TEA)_2_(FA)_4_Pb_5_I_16_		2.85	79.7% of PCE after 1080 h under environmental conditions (RT, RH = 30 ± 5%); without encapsulation	
	BA	(BA)_2_(MA)_49_Pb_50_Br_151_	FTO/TiO_2_/RPP/Spiro-OmeTAD/Au	9.5	60% of PCE after 30 h under harsh conditions of 1 sun illumination (90 °C and 50% humidity); without encapsulation	[196]
	PEA	(PEA)_2_(MA)_49_Pb_50_Br_151_		8.6	80% of PCE after 30 h under harsh conditions of 1 sun illumination (90 °C and 50% humidity); without encapsulation	
	PPA	(PPA)_2_(MA)_49_Pb_50_Br_151_		7.1	50% of PCE after 30 h under harsh conditions of 1 sun illumination (90 °C and 50% humidity); without encapsulation	
	PBA	(PBA_0.5_BA_0.5_)_2_MA_3_Pb_4_I_13_	ITO/PTAA/RPP/PCBM/BCP/Ag	16.0	93%, 85%, and 60% of PCE after 30 days for moisture, light, and heat aging, respectively	[69]
	n-BA	(n-BA)_2_MA_3_Pb_4_I_13_	FTO/C60/RPP/Spiro-OmeTAD/Au	5.38	N/A	[200]
	iso-BA	(iso-BA)_2_(MA)_3_Pb_4_I_13_		10.63	N/A	
	BA	(BA)_2_MA_3_Pb_4_I_13_	FTO/TiO_2_/RPP/Spiro-OmeTAD/Au	13.8	68.1% of PCE after 3500 h in a glovebox; without encapsulation	[97]
	BEA	(BEA)_2_MA_3_Pb_4_I_13_		16.1	83.1% of PCE after 3500 h in a glovebox; without encapsulation	
	BYA	(BYA)_2_MA_3_Pb_4_I_13_		15.1	77.2% of PCE after 3500 h in a glovebox; without encapsulation	

Through rationally employing these strategies to design organic spacers, RP PSCs have realized a significant improvement in PCE and stability. Nevertheless, there is still a large space room for further enhancement for commercialization. Future directions are presented as below:

1. More attempts should be made to develop novel organic spacer cations via the joint efforts from multiple strategies. These strategies are elaborated individually but they are always working jointly to affect the RP PSC performance. It is good to note that several studies do adopt multiple strategies to design spacer cations with encouraging results. For example, the typical case of TTFA and BThMA is achieved by combining strategies I and II [83], similarly 3,3-DFAz and DFP by combining strategies I and IV [31, 58], 5FPTMA by strategy I, strategy II and strategy IV and so on [100]. Adopting joint strategies to design organic spacers can maximize the roles of organic spacer to effectively modulate the properties of RPPs.
2. Most of current work is focusing on unary organic spacer. The design of new RPPs with binary organic spacers may bring in new hope to improve the photovoltaic technology. The synergistic effect of different organic spacers can further optimize the crystallization kinetics and regulate optoelectronic properties of RPPs. In this regard, future study should focus on revealing the cooperative mechanism of two or multiple organic spacers.
3. More promising results can be foreseen in future works, which can be achieved via multiple engineering methods such as solvent, antisolvent, additive, A-site and B-site composition alternation, or interface engineering. The film quality, phase spatial distribution, and energy level as well as charge transport properties at interfaces may not be in perfect conditions to match each other if only through organic spacer engineering method. Thus, multiple engineering methods should be jointly attempted to maximize the RP PSC performance and advance their development.
